# METTL3-dependent RNA m^6^A dysregulation contributes to neurodegeneration in Alzheimer’s disease through aberrant cell cycle events

**DOI:** 10.1186/s13024-021-00484-x

**Published:** 2021-09-30

**Authors:** Fanpeng Zhao, Ying Xu, Shichao Gao, Lixia Qin, Quillan Austria, Sandra L. Siedlak, Kinga Pajdzik, Qing Dai, Chuan He, Wenzhang Wang, James M. O’Donnell, Beisha Tang, Xiongwei Zhu

**Affiliations:** 1 Department of Pathology, Case Western Reserve University, 2103 Cornell Road, Cleveland, OH 44106 USA; 2 Department of Pharmaceutical Sciences, School of Pharmacy and Pharmaceutical Sciences, University at Buffalo, the State University of New York, Buffalo, NY 14214 USA; 3 Department of Neurology, Xiangya Hospital, Central South University, Changsha, Hunan China; 4 Department of Chemistry, The University of Chicago, Chicago, IL USA; 5 Department of Biochemistry and Molecular Biology, Howard Hughes Medical Institute, The University of Chicago, Chicago, IL USA

**Keywords:** RNA m^6^A modification, RNA methylation, METTL3, Alzheimer’s disease, Aberrant cell cycle events, Apoptosis

## Abstract

**Background:**

N6-methyladenosine (m^6^A) modification of RNA influences fundamental aspects of RNA metabolism and m^6^A dysregulation is implicated in various human diseases. In this study, we explored the potential role of RNA m^6^A modification in the pathogenesis of Alzheimer disease (AD).

**Methods:**

We investigated the m^6^A modification and the expression of m^6^A regulators in the brain tissues of AD patients and determined the impact and underlying mechanism of manipulated expression of m^6^A levels on AD-related deficits both in vitro and in vivo.

**Results:**

We found decreased neuronal m^6^A levels along with significantly reduced expression of m^6^A methyltransferase like 3 (METTL3) in AD brains. Interestingly, reduced neuronal m^6^A modification in the hippocampus caused by METTL3 knockdown led to significant memory deficits, accompanied by extensive synaptic loss and neuronal death along with multiple AD-related cellular alterations including oxidative stress and aberrant cell cycle events in vivo. Inhibition of oxidative stress or cell cycle alleviated shMettl3-induced apoptotic activation and neuronal damage in primary neurons. Restored m^6^A modification by inhibiting its demethylation in vitro rescued abnormal cell cycle events, neuronal deficits and death induced by METTL3 knockdown. Soluble Aβ oligomers caused reduced METTL3 expression and METTL3 knockdown exacerbated while METTL3 overexpression rescued Aβ-induced synaptic PSD95 loss in vitro. Importantly, METTL3 overexpression rescued Aβ-induced synaptic damage and cognitive impairment in vivo.

**Conclusions:**

Collectively, these data suggested that METTL3 reduction-mediated m^6^A dysregulation likely contributes to neurodegeneration in AD which may be a therapeutic target for AD.

**Supplementary Information:**

The online version contains supplementary material available at 10.1186/s13024-021-00484-x.

## Background

Alzheimer’s disease (AD) is a degenerative brain disorder and the most prevalent form of dementia with progressive synaptic dysfunction, neuronal loss and memory impairments [1]. Clinically expressed AD begins with the mild cognitive impairment (MCI) phase, which represents the earliest symptomatic stage to AD and other dementias [1–3]. Currently available FDA-approved treatments can only provide limited symptomatic alleviation. While more recent clinical trials are aimed at preventing disease progression in at-risk individuals or early stage in disease, none has been successful and many have been halted which is largely due to incomplete understanding of pathogenic mechanisms underlying synaptic dysfunction, neuronal loss and memory deficits in AD. Early onset AD which occurs in people before age of 65 is caused by mutations in APP, PS1 or PS2, but the cause of far more common late onset AD with age of onset older than 65 remains elusive. It is believed that a combination of genetics, environmental and lifestyle factors affect ones’ risk of developing AD [4]. Therefore, in addition to genetic studies, there are expanding number of studies on a potential role of epigenetic modifications in AD [5, 6]. These studies largely focused on epigenetic DNA modifications and provided important insights into the complex etiology of the late-onset AD.

Most recently, it has become clear that RNA modifications could also lead to alterations in gene expression [7] which allows extra layers of regulation of gene-environmental interaction. N6-methyladenosine (m^6^A) is the most common internal modification in multiple RNA species [8]. Reversible RNA m^6^A modification is dynamically modulated by the methyltransferases “writers” and demethylases “erasers” [9, 10]. RNA m^6^A is deposited by a multicomponent methyltransferase complex consisting of methyltransferase like 3 (METTL3) [11], METTL14 [12, 13], and Wilms Tumor 1 Associated Protein (WTAP) [14, 15]. Fat mass and obesity associated protein (FTO) [16] and α-ketoglutarate-dependent dioxygenase alkB homolog 5 (ALKBH5) [17] are responsible for m^6^A removal to achieve dynamic regulation. m^6^A modification exerts its effects by recruiting m^6^A -binding proteins to regulate RNA metabolism. It can be recognized by “readers” containing the YTH (YT521-B homology) domain [18], such as YTHDF1 [19], YTHDF2 [20] and YTHDF3 [21, 22]. A growing body of evidence indicates that RNA m^6^A methylation is involved in diverse biological processes including stress response regulation [23], adult neurogenesis [24], axon regeneration [25], synaptic function [26], and cognitive function [27–30]. Specifically, interrupting m^6^A-mediated regulation by knockdown of m^6^A readers in hippocampus altered synaptic gene expression and induced memory dysfunction [26, 29]. Meanwhile, reduction of m^6^A eraser FTO in hippocampus modulates learning process and memory formation in mice [27, 28]. The efficacy of hippocampus-dependent memory consolidation is regulated by METTL3 through promoting the translation of neuronal early-response genes [30]. However, the potential role of RNA m^6^A modification in AD and neurodegenerative diseases is largely unexplored.

In the present study, we showed that m^6^A-related regulators and neuronal RNA m^6^A modification were decreased in AD brains. Decreased m^6^A modification by METTL3 knockdown in the hippocampus in vivo resulted in elevated oxidative stress, aberrant cell cycle events, activation of apoptosis and neurodegeneration as well as cognitive deficits similar to that of AD. We found cell cycle inhibition or antioxidant could rescue METTL3 knockdown-induced neuronal deficits and neurodegeneration in vitro. Restored m^6^A modification by inhibiting its demethylation in vitro also rescued neuronal deficits and death induced by METTL3 knockdown. Thus, our results together suggest a potential role for RNA m^6^A dysregulation in the AD pathogenesis as well as its possible involvement in other neurodegenerative diseases.

## Materials and methods

### Postmortem brain samples

Cortical and hippocampal brain samples from postmortem AD and age-matched controls were collected at time of autopsy and stored at − 80 °C in the lab as described previously [31]. Brain samples were obtained under approved IRB protocols from the Brain Bank at Case Western Reserve University (CWRU). All AD cases were categorized based on clinical criteria established by CERAD and an NIA consensus panel [32, 33] and histopathologically confirmed according to the NINCDS-ADRDA criteria. Case information was provided in Table 1. Representative pictures of immunohistochemistry (IHC) for phosphorylated tau (using AT8 antibody) of an AD and control case were provided in Fig. S1A-C. No differences in post-mortem interval and ages between the AD and control tissues were observed in this study. The middle temporal gyrus samples from MCI and matched control cases were provided by Dr. Shi Jiong and Tom Beach (Barrow Neurological Institute). Human tissues were homogenized with RIPA lysis buffer for immunoblot analysis or fixed for immunohistochemical analysis.

**Table 1:**
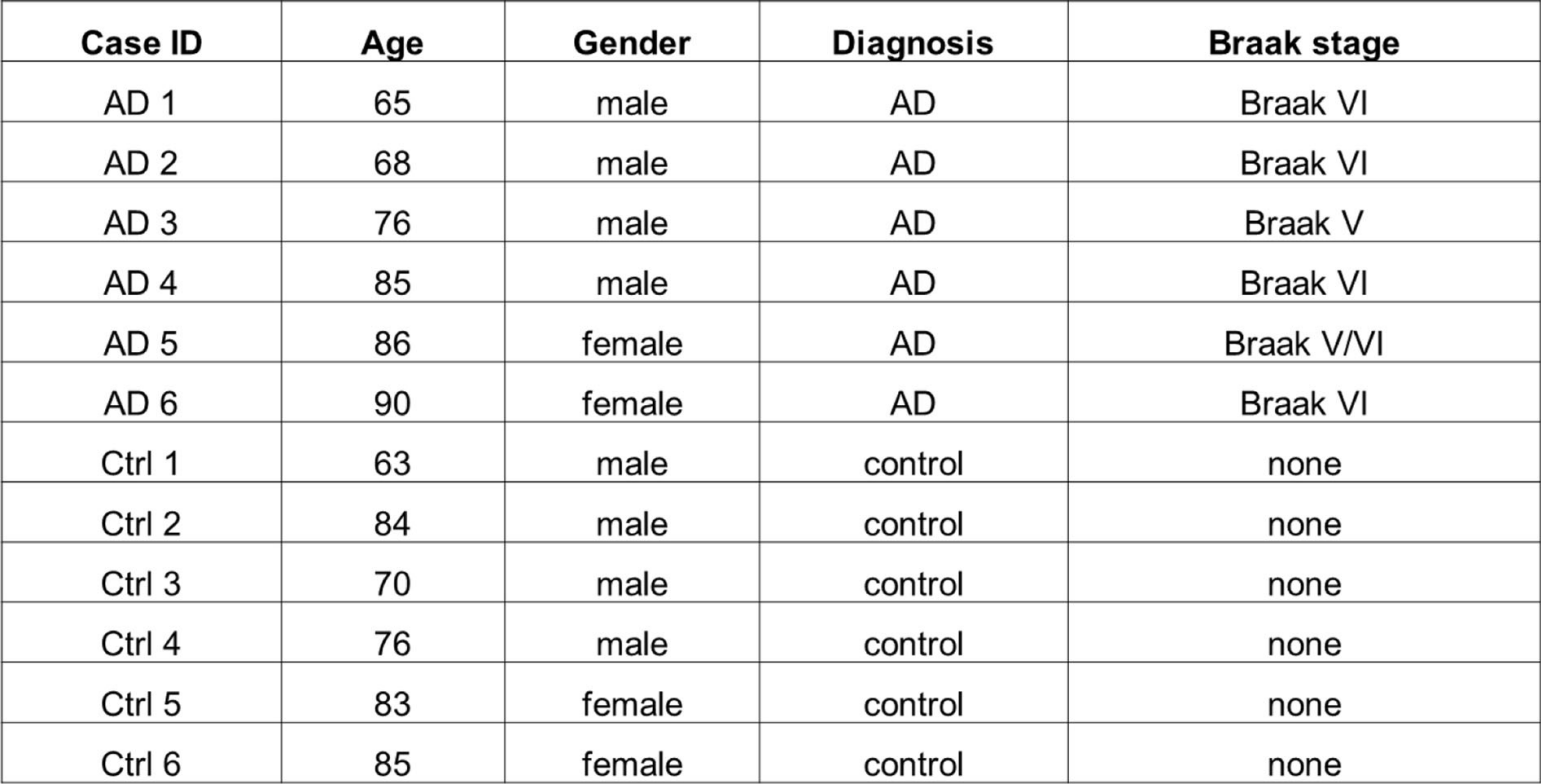
Clinical characteristics of AD and control cases

### Cell culture, plasmids, and treatment

Mouse N2a cells (CCL-131) were purchased from ATCC. The cells were grown in Opti-MEM I medium (Invitrogen, Carlsbad, CA, 31985088), supplemented with 5% (v/v) fetal bovine serum (Gibco, Carlsbad, CA, 10438026) and 1% penicillin-streptomycin (Gibco, Carlsbad, CA, 15140122). Primary neurons from E16 ~ 18 rat cortex (Charles River Laboratories) were seeded at 25000–40000 cells per well on coverslips or in 24-well plates coated with poly-D-lysine (Sigma, St. Louis, MO, P6407) in neurobasal medium (Gibco, Carlsbad, CA, 21103049) supplemented with 2% B27 / GlutaMAX (Gibco, Carlsbad, CA, 17504044 / 35050061). Half culture medium was changed every 3–4 days for primary neuronal cultures. All cultures were kept at 37 °C in a humidified 5% CO_2_-containing atmosphere. Soluble amyloid-β oligomers (AβOs) were prepared using Aβ_1–42_ peptide (California Peptide) as described before [31].

### Mice and surgery

Equal number of male and female wild-type C57BL/6 mice (000664) from Jackson Laboratory were used at 9–10 weeks of age for AAV infusion experiments and AβO treatment experiment. All procedures were approved by the Institutional Animal Care and Use Committee of CWRU (protocol# 2017–0028) and SUNY-Buffalo (protocol# PHC14093Y). All the mice were maintained under constant environmental conditions in the Animal Research Center with free access to food and water. Mice were randomly assigned to the various experimental groups and randomly selected for the stereotaxic infusion and behavioral experiments.

The shRNAs in this study were generated based on pAAV-U6-GFP expression vector (Cell Biolabs). The targeting sequence (GCTCTATCCAGGCCCATAA) of shRNA was designed for both mouse and rat Mettl3 mRNA and knockdown effect was verified in indicated cell lines. Nonfunctional shRNA (TAAGGTTAAGTCGCCCTCG) was used as a negative control. All the constructs were confirmed by DNA sequencing. The shRNA-Mettl3 (shMettl3) and negative control shRNA (shCtrl) were packaged into viruses for in vivo gene delivery by AAV helper free systems (Cell Biolabs) as previously described [34]. Briefly, AAV-293 cells were transfected with 3 plasmids: pHelper, pAAV-DJ Rep-Cap and pAAV-GFP-shRNA. Three days after transfection, cells were harvested and viruses were purified by AAV purification kit (6232, Clontech). Similarly, METTL3 coding sequence (plasmid #53739, addgene) was cloned to the AAV vector to generate AAV-METTL3 for overexpression and AAV-GFP was used as the control virus.

Animals were anesthetized using isoflurane and placed in a stereotactic frame. A small incision was made to expose the skull surface and to visualize bregma and holes were drilled in the skull overlying the dorsal hippocampus to deliver virus. One microliter virus (10^10^ infectious viral particles/mL) was stereotaxically infused bilaterally into the hippocampal at CA1 (− 2.1 mm anterior/posterior (A/P), 2.0 mm medial/lateral (M/L), 1.4 mm dorsal/ventral (D/V) from bregma) using a 10-μL microsyringe (Hamilton). Mice were allowed to recover for 3 weeks post-surgery before further behavioral tests. For AβO treatment experiment, a guide cannula was left in place and AβO was injected bilaterally two weeks after AAV-METTL3 infusion to the same spot [35, 36] and behavioral tests were performed two weeks later.

### Behavioral tests

All behavioral tests used equal numbers of male and female mice and were conducted 3 weeks after AAV infusion, starting with Y maze spontaneous alternation task (SAT) followed by open field test, novel object recognition task and object location test. The testing chamber and Y maze were cleaned after each trial to exclude olfactory cues. All of the behavioral tests were recorded and analyzed by EthoVision XT tracking system from Noldus.

#### Y maze

The animals were placed in a Y-shaped maze with 3 arms of equal length that diverge at equal angles and recorded for 5 min. The total number and sequence of arm entries over the test period was scored. An arm entry occurs when all 4 limbs are within the arm and a successful spontaneous alternation was defined as consecutive entries into all 3 arms without returning to a previously visited one. The maximal number of alternations was the total number of arm entries minus two, and the spontaneous alternation percentage was calculated as (actual alternations/maximal number of alternations) × 100.

#### Open field

For open field (OF) test, mice were placed in a light opaque chamber (50 cm × 50 cm) and recorded for 30 min. Total locomotor activity, time in center area, mean speed were automatically measured.

#### Object recognition tests

For novel object recognition (NOR) task, animals were placed in above chamber and allowed to explore 2 identical objects for 10 min. Two hours later, the animals were returned to the chamber with one of the two objects replaced by a novel object and the exploratory behavior was recorded for 5 min. The objects used in this study were T-25 cell culture flasks filled with sand and multicolored rectangular stacks. For object location test (OLT), spatial cues exist to help orient the mice during the training and test phases. In the training phase, two identical 50 mL falcon tubes filled with water were placed at adjacent far corners of the chamber, and mice were allowed to explore both objects in 3 blocks of 3 min each with 3-min breaks in between. Two hours later, the animals were returned to the chamber for test phase with one of the two objects displaced to adjacent empty corners, causing the 2 objects to be diagonal to each other. Mice were given 5 min to explore both objects, and the amount of time spent exploring each object was recorded. To analyze the cognitive performance, preference of novel object or displaced object was calculated as the time spent exploring the above objects divided by the cumulative time spent exploring both objects.

### Immunoblot

Brain tissues or neuronal cells were lysed in 1× radioimmunoprecipitation assay (RIPA) buffer (9806S; Cell Signaling Technology) containing protease inhibitor mixture (5,892,791,001 and 4,906,837,001, Roche). About 30 μg total protein extracts were resolved by SDS-PAGE and transferred to immobilon-P (IPVH00010, Millipore). After blocking with 10% nonfat milk in TBST, primary and secondary antibodies were applied and the blots were developed with Immobilon western chemiluminescent HRP substrate (WBKLS0500, Millipore). Quantification was performed using ImageJ.

Antibodies used in immunoblot were: rabbit anti-METTL3 (15073–1-AP, Proteintech), rabbit anti-METTL14 (ABE1338, Millipore), mouse anti-WTAP (60188–1-lg, Proteintech), mouse anti-FTO (MABE227, Millipore), rabbit anti-YTHDF1 (17479–1-AP, Proteintech), rabbit anti-YTHDF2 (24744–1-AP, Proteintech), rabbit anti-YTHDF3 (25537–1-AP, Proteintech), rabbit anti-GAPDH (2118, Cell Signaling), mouse anti-AT8 (MN1020, ThermoFisher), mouse anti-Actin (MAB1501, Millipore), mouse anti-γH2AX (ab26350, abcam), rabbit anti-PSD-95 (3409, Cell Signaling), mouse anti-synaptophysin (MA5–11475, Invitrogen), mouse anti-caspase 9 (sc-56,076, Santa Cruz), rabbit anti-cleaved caspase 3 (9661, Cell Signaling), mouse anti-CCND1 (sc-450, Santa Cruz), mouse anti-CCND2 (Santa Cruz, sc-56,305), mouse anti-CCNB1 (sc-245, Santa Cruz). Secondary antibodies included anti-mouse/rabbit HRP-linked secondary antibody (7076/7074, CST). Primary antibodies were used at a 1:1000 dilution, with secondary antibodies used at a 1: 10,000 dilution. All antibodies were validated for use in the study and detailed antibody validation profiles are available on the websites of the companies the antibodies were sourced from.

### Immunostaining

Immunohistochemical and immunofluorescent analyses were performed as described previously [37]. Briefly, brain tissues from human and mice were fixed and subsequently embedded in paraffin for sectioning. Brain sections (6 μm) were rehydrated and immunostained using primary antibodies as indicated. For immunohistochemistry, the sections were then incubated with either goat anti-mouse or goat anti-rabbit antibody, followed by species-specific peroxidase anti-peroxidase complex (Jackson, West Grove, PA, 223005024 or 323,005,024). 3–3′-Diaminobenzidine (Enzo Life Sciences, Farmingdale, NY, ACC105–0200) was used as a chromogen. All the images were acquired using Zeiss Axiovision software. For immunofluorescence, fixed neuronal cultures or rehydrated brain section were immunostained using the primary antibodies as indicated. Fluorescent secondary antibodies were subsequently applied and stained sections were imaged using Leica HyVolution SP8 confocal microscope at the CWRU SOM Light Microscopy Core Facility. Quantification was performed using ImageJ and adjacent blank area was selected as background in a region of interest for subtraction.

Antibodies used in immunostaining were: rabbit anti-METTL3 (15073–1-AP, Proteintech), rabbit anti-METTL14 (ABE1338, Millipore), mouse anti-FTO (MABE227, Millipore), rabbit anti-YTHDF1 (17479–1-AP, Proteintech), rabbit anti-m^6^A (202,003, Synaptic systems), mouse anti- m^6^A (NBP2–50525, Novus); mouse anti-AT8 (MN1020, ThermoFisher), mouse anti-NeuN (MAB377, Millipore), mouse anti-GFP (sc-9996, Santa Cruz), mouse anti-γH2AX (ab26350, abcam), rabbit anti-4-HNE (HNE11S, Alpha Diagnostic), rabbit anti-PSD-95 (3409, Cell Signaling), mouse anti-PSD-95 (sc-32,290, Santa Cruz), mouse anti-synaptophysin (MA5–11475, Invitrogen), rabbit anti-cleaved caspase 3 (9661, Cell Signaling), rabbit anti-MAP 2 (AB5622, Chemicon), rabbit anti-GFAP (PA5–16291, Invitrogen), mouse anti-GFAP (691,102, MP Biomedicals), rabbit anti-Iba1 (PA5–21274, Thermo Fisher), mouse anti-PCNA (sc-56, Santa Cruz), mouse anti-CCND1 (sc-450, Santa Cruz), mouse anti-CCND2 (Santa Cruz, sc-56,305), mouse anti-CCNB1 (sc-245, Santa Cruz). Other secondary antibodies included Alexa Fluor 488 donkey anti-mouse secondary antibody (A21202, Invitrogen), Alexa Fluor 568 goat anti-rabbit secondary antibody (A11036, Invitrogen), Alexa Fluor 568 goat anti-mouse secondary antibody (A-11004, Invitrogen), Alexa Fluor 647 goat anti-mouse (A21235, Invitrogen), Alexa Fluor 647 goat anti-rabbit (A21245, Invitrogen). Primary antibodies were used at a 1:200 dilution, with secondary antibodies used at a 1: 300 dilution.

### Golgi staining

Morphological alterations in dendritic spines in mouse brains were examined by Golgi staining using FD Rapid GolgiStain™ Kit (PK401, FD NeuroTechnologies) according to the manufacturer’s instructions. Spines were judged mushroom if the diameter of the head was much greater than the diameter of the neck as previously described [38].

### LC-MS/MS

100 ng RNA from human frontal cortex was digested with 1 μL nuclease S_1_ from *Aspergillus oryazae* (Sigma) in 50 μL reaction containing 10 mM NH_4_OAc pH 5.3 at 42 °C for 1.5 h. Then, 1 μL shrimp alkaline phosphatase (NEB), 3 μL 10 x CutSmart buffer (NEB) and 1 μL water were added and incubated at 37 °C for 1.5 h. After that, water was added to 50 μL and the samples were filtered with the 0.22 μm Millex Syringe filter (Millipore). 5 μL filtered sample was injected into a C18 column (Agilent) on a UHPLC (Agilent) coupled to a SCIEX 6500+ triple quadrupole mass spectrometer in positive electrospray ionization mode. The nucleotides were quantified based on the nucleoside-to-base transition 268➔136 (A), 282.1➔150.1 (m^6^A), the retention time 1.26 min (A), 2.6 min (m^6^A) and compared to the calibration curves.

### Reagents

Lipofectamine 2000 reagent (Invitrogen, 11,668,027) was purchased for transient transfection in neuronal cells according to the manufacturer’s instructions. Rnase (AM2286, Invitrogen) and Dnase (D4513, Sigma) treatments were performed according to the manufacturer’s instructions. Cresyl violet acetate (C5042, Sigma) 2.5% aqueous is used in Nissl staining of mouse brain sections. Actinomycin D (A1410, Sigma), N-Acetyl Cysteine (NAC, A7250, Sigma), flavopiridol (10,009,197, Cayman) and rhein (17,345, Cayman) were purchased and used in the treatment of primary neurons as indicated. In Situ Cell Death Detection Kit (11,684,795,910, Roche) was used to label rehydrated mouse brain sections undergoing apoptosis following manufacturer’s instructions in this study. This method is based on labeling of DNA strand breaks (TUNEL technology) and analyzed by fluorescence microscopy. After TUNEL labeling, the sections were stained for GFP using Alexa Fluor 568 goat anti-mouse secondary antibody (A-11004, Invitrogen) and FITC-labelled cells were imaged on Leica HyVolution SP8 confocal microscope.

### Measurements in primary neurons

Data showing incidence of events in primary cortical neuronal cells (dendritic spine number and neurite degeneration) were obtained from multiple random regions from three independent experiments. Distal neurites of primary neurons were defined to be shortened if the neurites did not extend a 40× microscopic field of view as previously described [39]. Propidium iodide (P3566, Invitrogen) was added to the AAV-GFP-shRNA-infected primary neuronal cultures for 4 h before measurements in fluorescence microscopy.

### Quantitative PCR analysis

To detect cellular mRNA changes in primary rat cortical neurons, RNA samples were reversely transcribed using 6-nucleotide random primers and M-MLV reverse transcriptase (M0253, NEB). Quantitative PCR reactions were performed with SYBR™ Green Master Mix (43–856-12, Applied Biosystems) and specific primers on the StepOne 96-well Real-Time PCR System (Applied Biosystems). The mRNAs levels were normalized to the GAPDH gene or 18SrRNA as indicated. The PCR reaction was performed at 95 °C for 5 min followed by 45 cycles at 95 °C for 10 s, 60 °C for 30 s. Each set of PCR reaction was performed in triplicate and CT values of each PCR reaction were obtained. The ratios of gene expression were calculated relative to the control set in the experiments. The following primer pairs were used: rat GAPDH, 5′-CAA GGA GTA AGA AAC CCT GGA C-3′ (sense) and 5′-GGG ATG GAA TTG TGA GGG AGA T-3′ (antisense); rat CCNA1, 5′- TGA ACA GGG GGA CAG AGA CA-3′ (sense) and 5′- GAG TCA ACC AGC ATT GGG GA-3′ (antisense); rat CCNB1, 5′- TCC CAC ACG GAG GAA TCT CT-3′ (sense) and 5′- TCT GCA GAC GAG GTA GTC CA-3′ (antisense); rat CCND1, 5′- TCA AGT GTG ACC CGG ACT G-3′ (sense) and 5′- CAC TAC TTG GTG ACT CCC GC-3′ (antisense); rat CCND2, 5′- GCT CTG TGT GCT ACC GAC TT − 3′ (sense) and 5′- GGT CCG GAT CTT CCA CAG AC − 3′ (antisense); rat CDKN1C 5′- GAA CGG TGC GAT CAA GAA GC − 3′ (sense) and 5′- ATG AAA GGT CCC AGC CGA AG − 3′ (antisense). Rat 18SrRNA 5′- GCT TAA TTT GAC TCA ACA CGG GA − 3′ (sense) and 5′- AGC TAT CAA TCT GTC AAT CCT GTC − 3′ (antisense).

### Anti-m^6^A immunoprecipitation

Immunoprecipitation (IP) of m^6^A-modified RNA using a specific mouse anti-m^6^A (NBP2–50525, Novus) was carried out following a published protocol [40]. Briefly, total pure RNA (1.5 μg) was treated by DNase and fragmented, followed by incubation with m^6^A antibody (2 μg)-Protein G-Magnetic Beads (1.5 mg) (MJS002V2, MBL) complex in 1 × RNA IP buffer (150 mM KCl, 25 mM Tris pH 7.4, 5 mM EDTA, 0.5 mM DTT, 0.5% IGEPAL® CA-630). After extensive washing with 1 × RNA IP buffer, RNA fragments with m^6^A modification were eluted using elution buffer with free m^6^A 5′monophosphate sodium salt (NM10586, Carbosynth). Eluted RNA was used to synthesize cDNA for quantitative PCR analysis to detect the m^6^A-modified target mRNA. Same amount of fragmented pure total RNA was saved as input reference. Normal mouse IgG_1_ (2 μg) (sc-3877, Santa Cruz) was used as the negative control antibody in the m^6^A IP experiment. All of the solutions were treated by RNAsecure (AM7006, Invitrogen) to irreversibly inactivate RNases. Following primers were used to detect the potential m^6^A modified regions in 3’UTR of the target genes using quantitative PCR analysis: CCND2 sense, 5′-TAG TGA GAT GCT TAC AGG A-3′ and CCND2 antisense, 5′-TGC TTT GCA AAC TAC TCA TGC G-3′.

### Statistical analysis

The performer(s) was (were) blinded to the experimental design in data collection and analysis. All the data represent means± standard error of the mean (SEM). For 2 independent data comparisons, two-tailed unpaired student’s t-test was used to determine statistical significance. For multiple comparisons, one-way ANOVA with bonferroni’s correction was used as indicated in the text. All the statistical analyses were performed using Graphpad Prism or Excel 2016. *P*-values < 0.05 were considered significant. Data distribution was assumed to be normal, but this was not formally tested. Clincalc was used to predetermine the minimum number sample sizes of mouse behavioral tests.

## Results

### Decreased neuronal m^6^A modification in AD brains

**Fig. 1:**
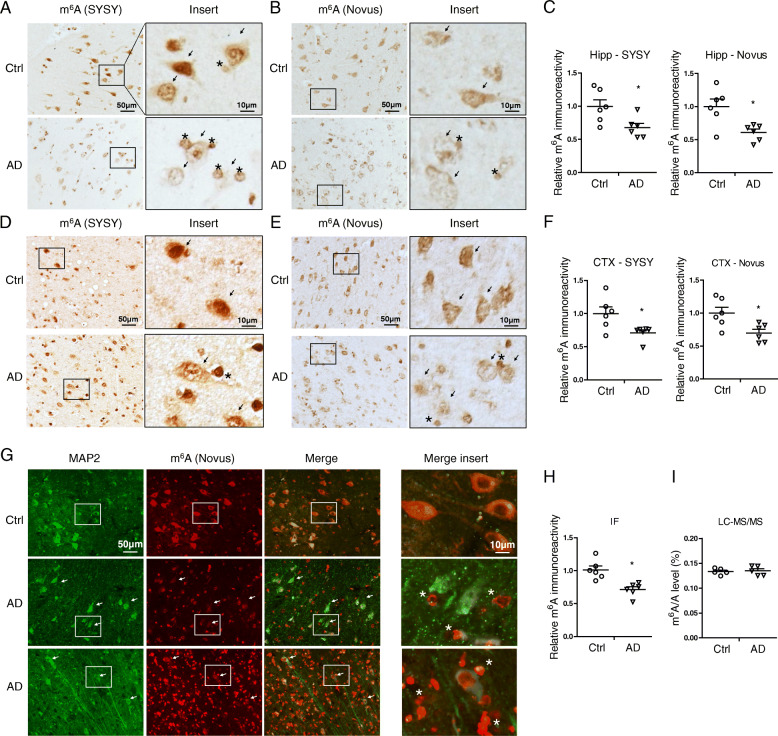
Decreased neuronal m6A modification in AD brains. **(A-F)** Representative immunohistochemical staining (A-B, D-E) and quantifications (C,F) of m6A using two different antibodies [rabbit antibody from SYSY (A, D) and mouse antibody from Novus (B, E)] in hippocampus (Hipp) (A-C) and cortex (CTX) (D-F) from human AD and age-matched control cases. **(G-H)** Colocalization analysis of m^6^A with neuronal marker MAP 2 in hippocampal tissues from AD and control brains (G) and quantification of neuronal m^6^A immunoreactivity colocalized with MAP 2 was shown (H). (I) Global m^6^A level was measured using total RNA isolated from AD and control frontal cortical tissues by LC-MS/MS. The arrows in inserts (A-G) point to neurons and asterisks indicate glial cells (*n* = 5–6 cases per group). (Data are means±SEM, *p < 0.05, (C, F, H, I) unpaired student’s t-test)

To elucidate a potential role of m^6^A modification during the course of AD, we first measured the level of m^6^A immunoreactivity in the hippocampus and cortex from AD and age-matched control patients. The immunoreactivity of m^6^A was found primarily in pyramidal neurons throughout the hippocampus (Fig. 1 A,B,C) and cortex (Fig. 1 D,E,F) of human brains using two different antibodies. Some smaller cells resembling glial cells were occasionally stained. While both DNA and RNA can have m^6^A modifications [41, 42], neuronal m^6^A immunoreactivity was only dramatically reduced after Rnase treatment but not after Dnase treatment (Fig. S1 D), indicating that m^6^A modification predominantly exists in RNA species in human brain tissues. Comparing to m^6^A immunostaining in age-matched control brains, the RNA m^6^A modification was significantly reduced in large pyramidal neurons in the AD brain (Fig. 1 A-F). Meanwhile, it was noted that m^6^A immunoreactivity was increased in some glial-like cells surrounding the neurons in AD brain tissues (Fig. 1 A-F). No cellular structures were noted by secondary only negative controls in all of human cases from immunohistochemical and immunofluorescent analyses (Fig. S1 E-F and S2 E). Immunofluorescence co-localization study with neuronal and glial cell markers confirmed that m^6^A immunoreactivity was dramatically reduced in MAP 2-positive neurons (Fig. 1 G,H) but increased in GFAP-positive astrocytes (Fig. S1 A-C) and some iba-1 positive microglia (Fig. S1 D) in AD brains. However, the total m^6^A level was unchanged in AD brains compared with normal controls as measured by LC-MS/MS (Fig. 1 I), likely caused by differentially changed m^6^A levels in neurons and glial cells.

### Decreased m^6^A-related regulator proteins in AD brains

**Fig. 2:**
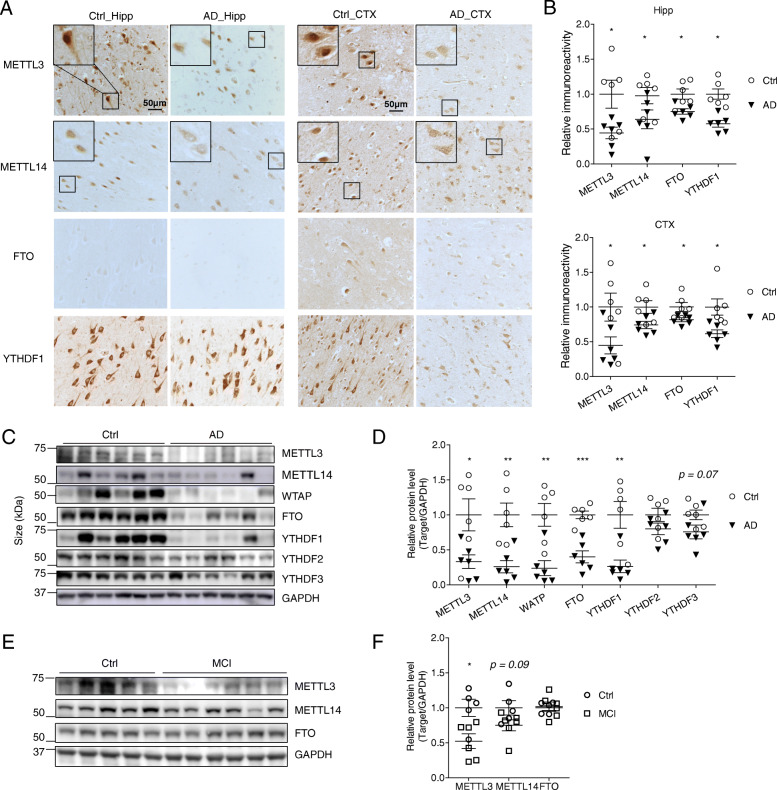
Decreased m^6^A-related regulator proteins in AD brains. **(A-B)** Representative immunohistochemical staining (A) and quantifications (B) of METTL3, METTL14, FTO or YTHDF1 in hippocampus (Left, Hipp) and cortex (Right, CTX) from human AD and age-matched control cases (*n* = 6 cases per group). **(C-D)** Representative immunoblot (C) of proteins of m^6^A regulators in frontal cortical tissues from human AD and age-matched control cases and quantitative analysis (D) revealed significantly decreased protein levels of many of these regulators in AD brains (n = 6 cases per group). **(E-F)** Representative immunoblot (E) of proteins of m^6^A regulators in middle temporal gyrus tissues from human MCI and age-matched control cases and quantitative analysis (F) revealed significantly decreased METTL3 protein levels in MCI brains (n = 5–6 cases per group). GAPDH was probed as an internal loading control. (Data are means±SEM, *p < 0.05, **p < 0.01, ***p < 0.001; (B, D, F) unpaired student’s t-test)

RNA m^6^A modification is dynamically modulated by m^6^A writers and erasers and recognized by m^6^A readers [43]. Protein expression of m^6^A writers (METTL3, METTL14, and WTAP), eraser (FTO) and readers (YTHDF1, YTHDF2, and YTHDF3) in brain tissues from AD patients and age-matched control patients was determined by both immunocytochemistry and immunoblot analysis. Immunocytochemical analysis revealed extensive immunoreactivities of METTL3 and METTL14 throughout the cell body but more predominantly in the nucleus in pyramidal neurons in the hippocampus and cortex (Fig. 2 A) from elder controls. However, in AD brain, reduced immunoreactivities of METTL3 and METTL14 were noted in these neurons with a more uniform distribution between the cytosol and nucleus (Fig. 2 A,B). The m^6^A eraser FTO was observed both in cytosol and nucleus in the pyramidal neurons from controls (Fig. 2 A,B) but only background immunoreactivity of FTO was noted in these neurons from AD patients. The m^6^A reader YTHDF1 existed predominantly in the cytosol of pyramidal neurons in age-matched control brains but with reduced immunoreactivity in AD brains (Fig. 2 A,B).

Consistently, immunoblot analysis demonstrated significant reduction in the protein levels of many of these m^6^A regulators in the brain cortical tissues from AD patients compared to that from age-matched control patients (Fig. 2 C,D): the m^6^A writers, including METTL3, METTL14 and WTAP, were significantly reduced by 66.7, 74.0, and 76.0% in AD, respectively. The eraser FTO was also significantly reduced by 60% in AD. Among the three readers measured, the level of YTHDF1 protein was significantly reduced by 73.5% while levels of YTHDF2 or YTHDF3 proteins were reduced but did not reach significance in AD.

MCI represents the earliest symptomatic stage to AD [2, 3]. Middle temporal gyrus is one of the first temporal lobe neocortical sites affected in AD, and atrophy in middle temporal gyrus may herald the presence of future AD [44]. To investigate whether m^6^A modification plays a role in the early stage of AD disease development, we further measured the protein levels of m^6^A writers and eraser in the middle temporal gyrus from patients with MCI (Fig. 2 E). Immunoblot analysis showed that only METTL3, but not METTL14 and FTO, was significantly reduced in middle temporal gyrus from MCI patients compared with that from normal controls (Fig. 2 E,F). These data suggest that dysregulation of RNA m^6^A modification is likely involved in the pathogenesis of AD early in the process and METTL3 reduction likely plays a critical role.

### Decreased neuronal METTL3 and m^6^A in the hippocampus by AAV-shMettl3

**Fig. 3:**
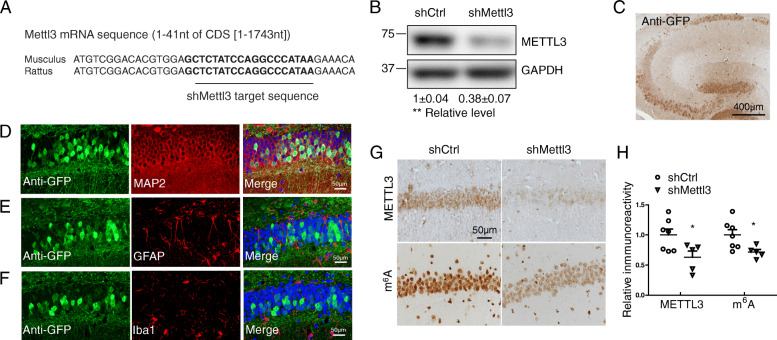
Decreased neuronal METTL3 and m^6^A in the hippocampus by AAV-shMettl3. **(A)** Highly conserved gene sequences of shRNA targeting region in mouse and rat Mettl3 mRNA. **(B)** Knockdown effect of shMettl3 was confirmed in mouse N2a cells 3 days after transient transfection (Data are means±SEM from 3 independent experiments). **(C)** AAV-mediated gene delivery in the hippocampus was confirmed by immunostaining for GFP. WT C57BL/6 mice receiving bilaterally stereotaxic injections of AAV encoding for eGFP plus anti-Mettl3 shRNA (shMettl3) or scrambled control shRNA (shCtrl) into the hippocampus at 2 months of age were analyzed 1 month later. **(D-F)** Representative images of the AAV-infected areas (GFP-positive) in hippocampus for different cell markers by immunocytochemistry after fixation: MAP 2 for neurons (D), GFAP for astrocytes (E), and Iba1 for microglia (F). **(G, H)** Representative image of immunoreactivity of METTL3 or m^6^A (G) in AAV-shRNA-injected mice and quantification analysis (H) confirmed that METTL3 protein and m^6^A modification level were reduced in mice injected with shMettl3 virus (n = 5–7 mice per group). GFP staining has been performed in adjacent brain slices at the same time and immunoreactivities of m^6^A and METTL3 were measured in GFP-positive regions. (Data are means±SEM, *p < 0.05, **p < 0.01, (B, H) unpaired student’s t-test)

To determine the potential role of METTL3 downregulation and disturbed m^6^A regulation in neurodegeneration and pathogenesis of AD, we sought to knockdown the neuronal expression of METTL3 in vivo by using adeno-associated virus (AAV) containing vectors co-expressing enhanced green fluorescent protein (eGFP) and METTL3-targetting shRNA (shMettl3) and determine AD-related cognitive and pathological changes. Specifically, the shMettl3 targeting sequence is located in a highly conserved region in both mouse and rat METTL3 mRNAs (Fig. 3 A), which allows efficient METTL3 knockdown in both mouse and rat neuronal cells. Significant reduction of endogenous METTL3 protein level was confirmed by western blot analysis in mouse neuroblastoma N2a cells (Fig. 3 B) and rat primary neurons (not shown) transiently transfected with AAV vector containing shMettl3 as compared to cells transfected with AAV vector containing a scramble shRNA as a negative control (shCtrl).

Wild type mice were bilaterally injected with AAV-GFP-shRNA (shMettl3 or shCtrl) into the hippocampal area at 9–10 weeks of age, and sacrificed and analyzed 4 weeks later. Needle tracks of intracranial injections were validated to confirm proper AAV infusion in hippocampus and no clear cell loss was noted around injection sites in all of the shCtrl mice (Fig. S3 A). Immunocytochemical analysis of GFP expression revealed GFP-positive cells mainly in the CA3 and CA1 regions in both shCtrl and shMettl3-infuced mice (Fig. 3 C). Pyramidal neurons in the hippocampus were preferentially infected as confirmed by the predominant co-localization of GFP and neuronal marker MAP 2 (Fig. 3 D), but little or no colocalization of GFP and the astrocytic marker GFAP (Fig. 3 E) or the microglial marker Iba1 (Fig. 3 F). Consequently, immunocytochemical analysis confirmed that endogenous METTL3 proteins along with neuronal m^6^A level were significantly reduced in GFP-positive pyramidal neurons of shMettl3-injected mice compared to that of shCtrl-injected mice (Fig. 3 G,H), confirming the major role of METTL3 in m^6^A modification in pyramidal neurons in the adult hippocampus.

### METTL3 knockdown in the hippocampus leads to memory loss

**Fig. 4:**
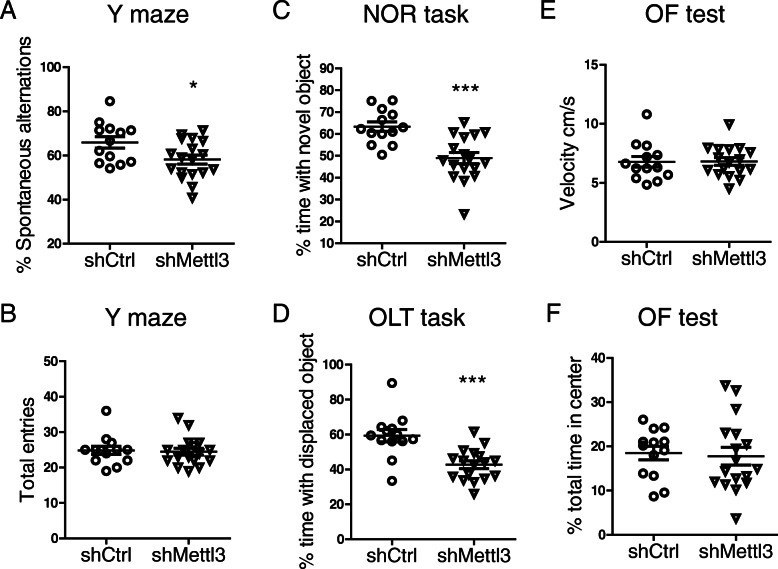
METTL3 knockdown in the hippocampus caused cognitive/memory deficits. **(A, B)** showed percent of spontaneous alternations (A) and total entries (B) in the Y-maze assay for shMettl3-injected WT mice and shCtrl-injected mice. **(C-D)** The NOR task (C) was used to assess recognition memory and the OLT task (D**)** was used to assess the hippocampus-dependent spatial memory function in shRNA-injected mice. The preference index for each mouse was determined by dividing the amount of time spent exploring the novel (C) or displaced (D) object by the total amount of time spent exploring both objects. **(E, F)** Total distance traveled and time in the center in the open field (OF) test were recorded. Locomotor activity (E) and percent total time in center (F) were calculated. (*n* = 13 mice in shCtrl-injected group; *n* = 17 mice in shMettl3-injected group). (Data are means±SEM, *p < 0.05, ***p < 0.001; (A-F) unpaired student’s t-test)

Several behavioral tests were carried out 3 weeks after intracranial injections. In the Y-maze spontaneous alternation task (SAT), shMettl3-injected mice exhibited reduced spontaneous alternation compared to shCtrl-injected mice (Fig. 4 A), indicating an impairment in the spatial working memory caused by the knockdown of METTL3 in the hippocampus. In the novel object recognition (NOR) task to assess mouse ability to explore longer a novel object over a familiar one (Fig. 4 C), shCtrl-injected mice exhibited a 63% preference indices for the novel object as expected. In contrast, shMettl3-injected mice exhibited no differences in the exploration time between novel and familiar objects, indicating an impairment in recognition memory. The hippocampus-dependent spatial memory was further evaluated using the object location test (OLT) which also revealed significantly decreased percentage of time interacting with the displaced object in shMettl3-injected mice (Fig. 4D). shMettl3 mice and shCtrl mice did not differ in the total entry number of arms during SAT test (Fig. 4 B) and the amount of locomotor activity during open field (OF) test (Fig. 4 E), suggesting that METTL3 reduction in the hippocampus did not affect motor performance. No anxiety-like behaviors were observed in either shCtrl mice or shMettl3 mice, as evidenced by the same amount of time spent in the center during OF test (Fig. 4 F). Together, these results suggest decreased neuronal m^6^A modification in the hippocampus by METTL3 knockdown leads to cognitive deficits, the cardinal feature of AD.

### METTL3 knockdown in the hippocampus causes neurodegeneration, spine loss and gliosis

**Fig. 5:**
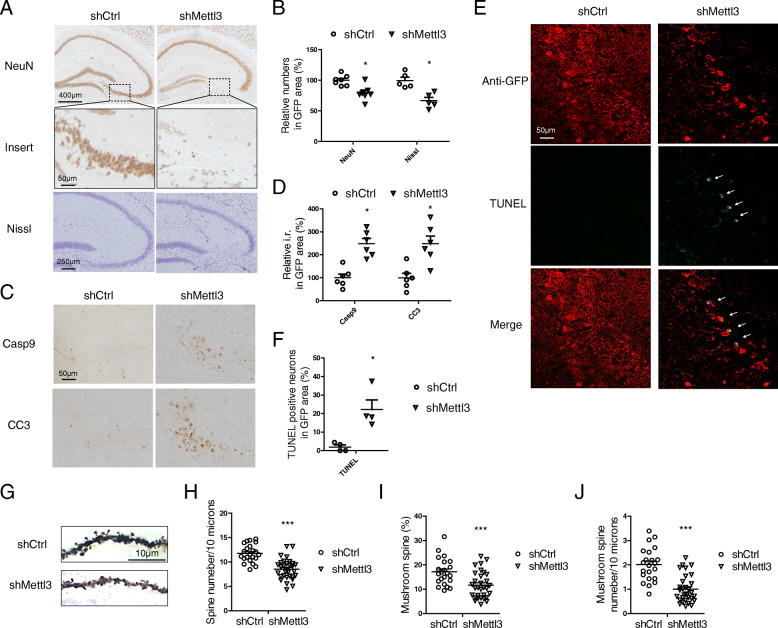
METTL3 knockdown in the hippocampus caused neurodegeneration and dendritic spine loss. **(A-B)** Representative immunohistochemistry images (A) of NeuN or cresyl violet staining (Nissl staining) in the hippocampus of shRNA-injected mice and quantifications (B) of relative cell numbers in GFP-positive area. **(C-D)** Representative immunohistochemistry images (C) and quantifications (D) of caspase 9 and cleaved caspase 3 in the GFP-positive hippocampal regions of shRNA-injected mice. **(E-F)** TUNEL staining (Excitation laser wavelength 488 nm) was performed in GFP (Alexa Fluor 568) stained hippocampal sections from shRNA-injected mice (E) and quantifications of relative TUNEL-positive cell numbers in GFP-positive regions were shown (F). **(G-J)** Golgi staining was performed to reveal the spine structures in hippocampal CA1 neurons 4 weeks after AAV-GFP-shRNA injection (G). Quantification shows that dendritic spine number (H) and mushroom spine number (I, J) in CA1 neurons are decreased by METTL3 depletion. (A-F, *n* = 4–6 mice in each group; H-J, 22–31 random dendrites from 4 mice were analyzed) (Data are means±SEM, *p < 0.05, ***p < 0.001, B, D, F, H-J unpaired student’s t-test)

AD patients suffer from extensive selective neuronal loss in the hippocampal areas [45, 46]. Interestingly, dramatic neuronal loss was found in the hippocampus of shMettl3-injected mice compared with that of shCtrl-injected mice as evidenced by the significant loss of NeuN-positive neurons. This was further confirmed by the significant loss of Nissl-stained cells (Fig. 5 A,B). Caspase 3 is a key mediator of neuronal programed cell death and its activation is a feature of many neurodegenerative diseases [47, 48]. Indeed, increased immunoreactivities of activated/cleaved caspase 3 and its upstream activator, caspase 9, were found in the hippocampus of shMettl3-injected mice as compared to that of shCtrl-injected mice (Fig. 5 C,D). TUNEL staining also revealed the appearance of apoptotic cells in the hippocampal region of the shMettl3-injected mice but not in the shCtrl-injected mice (Fig. 5 E,F). To assess neuronal deficits, Golgi staining was performed to investigate synaptic changes (Fig. 5 G). Significantly decreased number of dendritic spines (Fig. 5 G,H) and stable mushroom spines (Fig. 5 G,I,J) were found in the pyramidal neurons in the hippocampus of shMettl3-injected mice which likely underlies cognitive deficits in these mice.

In addition to neuronal deficits, increased activation of microglia (Fig. S3 B,C) detected by iba-1 immunostaining and increased activation of astrocytes (Fig. S3 D,E) detected by GFAP staining were also found in the hippocampus of shMettl3-infected mice compared with shCtrl-injected mice. Considering that AAV-shRNA only infected neurons in the hippocampus (Fig. 3 D-F), the increased microgliosis and astrocytosis is likely a response to neuronal damage.

### METTL3 knockdown in the hippocampus causes oxidative stress and aberrant cell cycle events

**Fig. 6:**
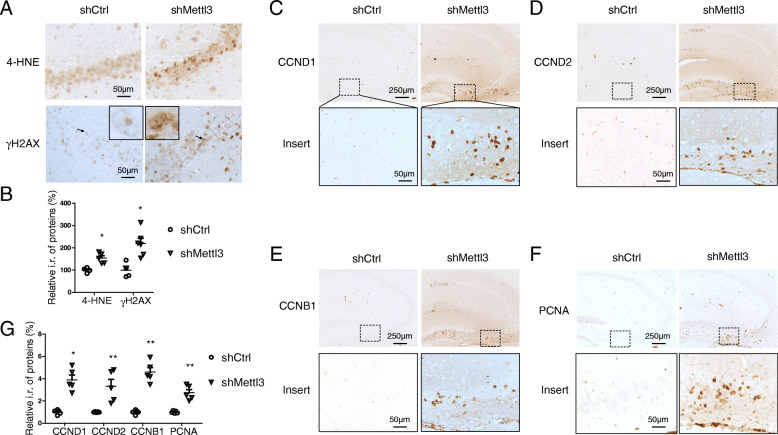
METTL3 knockdown in the hippocampus caused oxidative stress and aberrant cell cycle events. **(A-G)** Representative immunohistochemistry images of 4-HNE (A, upper), γH2AX (A, lower), CCND1 (C), CCND2 (D), CCNB1 (E) and PCNA (F) in hippocampal CA3 region in shRNA-injected mice. Quantifications showed increased immunoreactivities of above oxidative stress makers (B) and cell cycle makers (G) by METTL3 depletion in the hippocampus. (n = 4–6 mice in each group.) (Data are means±SEM, *p < 0.05, **p < 0.01, B, G unpaired student’s t-test)

Extensive oxidative stress features the vulnerable regions of the brain affected by AD and it is considered to be critical to AD pathogenesis [49]. The immunoreactivity of 4-hydroxy-2-trans-nonenal (HNE), an oxidative stress marker, was significantly increased in the hippocampus of shMettl3-injected mice (Fig. 6 A,B). Continuous attack of DNA by reactive oxygen species (ROS) leads to DNA damages such as strand breaks [50]. Consistently, increased DNA damage, as demonstrated by significantly increased immunoreactivity of γ-H2AX (Fig. 6 A,B), was also found in the hippocampus of shMettl3-injected mice.

Aberrant cell cycle activation is a critical component of the DNA damage response in postmitotic neurons [51]. Unexpected neuronal death can be induced by aberrant cell cycle events in postmitotic neurons in the central nerve system [52] which is believed to be involved in neuronal loss in AD [53, 54]. Interestingly, increased immunoreactivity of cyclin-D1 (CCND1), cyclin-D2 (CCND2) and cyclin B1 (CCNB1) was found in pyramidal neurons in the hippocampus by METTL3 depletion, but none of these cyclins were observed in shCtrl mice (Fig. 6 C-E, G). Abnormal expression of proliferating cell nuclear antigen (PCNA), one of the cell cycle proteins, was reported to predict the sites of neuronal cell death in AD brains [55] and animal models of neurodegeneration [56, 57]. PCNA-positive cells were also found in the hippocampus of shMettl3-injected mice, but not in shCtrl-injected mice (Fig. 6 F,G). Our data suggest that METTL3 knockdown causes increased oxidative stress and aberrant cell cycle events, which could be involved in METTL3 knockdown-induced neuronal death.

### METTL3 knockdown induces dysregulation of cell cycle genes and apoptotic changes in primary neuronal cultures

**Fig. 7:**
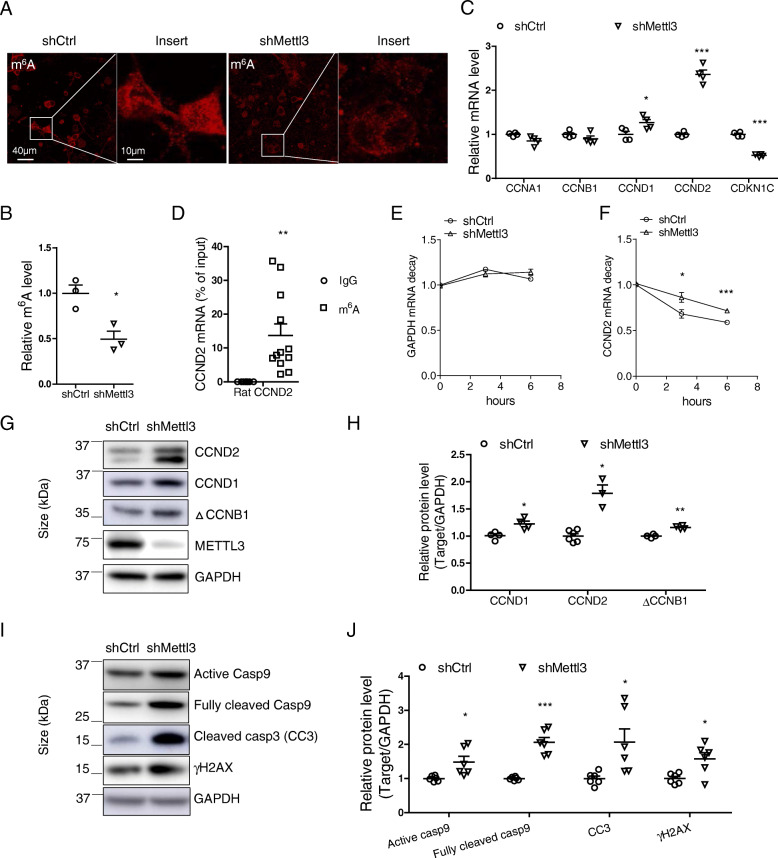
METTL3 knockdown induced dysregulation of cell cycle genes and apoptotic changes in primary neuronal cultures. **(A, B)** Representative immunofluorescence image (A) and quantification analysis of m^6^A immunoreactivity (B) in primary cortical neurons after AAV-GFP-shRNA infection revealed significantly decreased neuronal m^6^A modification by METTL3 depletion. **(C)** Quantitative PCR analysis of cell cycle genes in primary neurons after Mettl3 knockdown mediated by AAV-shRNA infection. GAPDH was used as a reference gene for quantitative gene expression analysis. **(D)** Total pure RNA from adult rat cortical tissue was immunoprecipitated with a specific m^6^A antibody (Novus) or a mouse IgG control. CCND2 mRNAs were pulled down and detected by quantitative PCR. **(E-J)** Primary rat cortical neurons from E16–18 embryos were infected with AAV-GFP-shMettl3 or AAV-GFP-shCtrl on DIV 7. (E-F) Six days after AAV injection, neurons were treated with actinomycin D (5 μg/ml). Representative mRNA profiles of GAPDH (E) and CCND2 (F) at 0-, 3-, and 6-h time points after treatment were shown. 18S rRNA was used as reference for quantitative analysis. (G-J) Eight to nine days after AAV infection, neuron extracts were used for immunoblot analysis (G, I) and quantification of protein changes were shown (H, J) (Data are means±SEM from at least 3 independent experiments, *p < 0.05, **p < 0.01, ***p < 0.001; B-F, H, J unpaired student’s t-test)

To determine the relationship between METTL3 knockdown-induced aberrant cell cycle events and neurodegeneration, we investigated cell cycle changes in primary neurons after METTL3 knockdown in vitro. Transfection of shMettl3 effectively reduced endogenous METTL3 protein level (Fig. S4 A,B) and m^6^A immunoreactivity in rat primary neurons (Fig. 7 A,B). Analysis of expression level of multiple cyclin genes assessed by quantitative PCR analysis (Fig. 7 C) revealed that METTL3 knockdown led to significantly elevated mRNA levels of CCND1 and CCND2 in primary neurons. Interestingly, cyclin dependent kinase inhibitor 1C (CDKN1C) was dramatically decreased by METTL3 knockdown (Fig. 7 C). It is worth noting that CCND2 was identified as a m^6^A target in the mouse brain [58] and we also verified by m^6^A-immunoprecipitation (IP) assay that m^6^A antibody pulled down about 12% of total input CCND2 mRNA by quantitative PCR (Fig. 7 D). To examine whether increased gene expression was caused by changes in m^6^A-mediated RNA decay, we measured the life time of CCND2 mRNA by inhibition of gene transcription with actinomycin D in primary cortical neurons. After actinomycin D treatment, GAPDH mRNA level was not apparently affected in either shCtrl neurons or shMettl3 neurons at 3 and 6 h (Fig. 7 E). However, CCND2 mRNA level was declined by 32 and 41% in shCtrl neurons at 3 and 6 h after treatment, respectively. In the contrary, CCND2 mRNA was declined by only 14 and 28% in shMettl3 neurons (Fig. 7 F). Our data indicated mRNA level of CCND2 in shCtrl neurons declined more rapidly than in shMettl3 neurons after inhibition of gene transcription. Thus, the increased level of m^6^A-modified CCND2 by the depletion of METTL3 was likely caused by delayed mRNA clearance. Consistent with mRNA changes, immunoblot analysis revealed that the protein levels of CCND1 and CCND2 were significantly increased in primary neurons by shMettl3 (Fig. 7 G,H). Interestingly, METTL3 depletion caused significant increase in ~ 35 kDa protein product of CCNB1 (△CCNB1) (Fig. 7 G,H), a caspase-dependent cleavage product during mitotic catastrophe sufficient to induce mitotic block and apoptosis [59]. Indeed, Caspase-9/3 apoptotic pathway was activated as evidenced by increased active/cleaved caspases and increased DNA damage response was also detected as indicated by increased γH2AX after METTL3 treatment in these primary neurons (Fig. 7 I,J).

### METTL3 knockdown causes postsynaptic deficit and neurite degeneration in primary neuronal culture

**Fig. 8:**
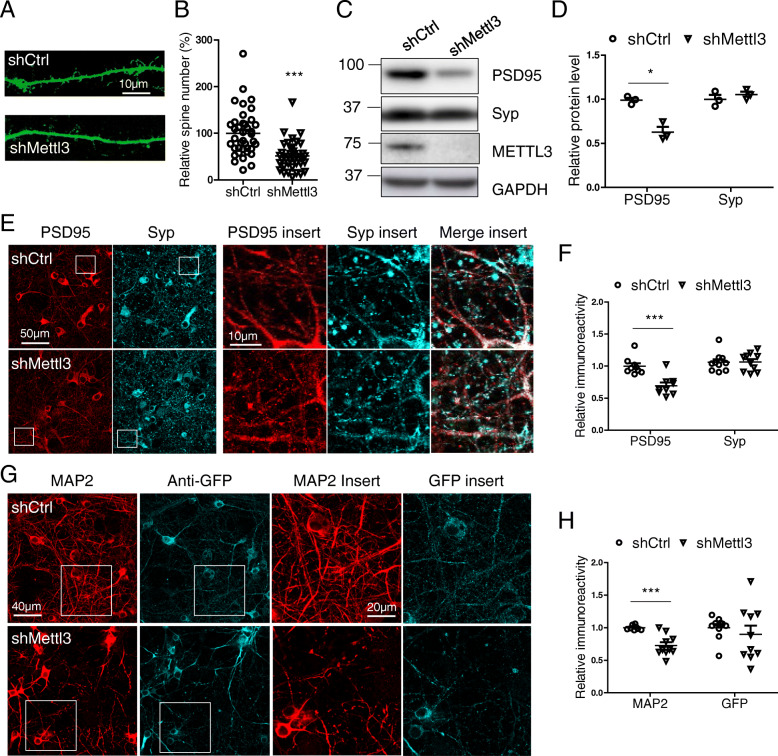
METTL3 knockdown caused postsynaptic damage and neurite degeneration in primary neuronal culture. **(A, B)** Primary cortical neurons were transfected with GFP-shRNA at DIV12. Dendritic spine morphology (A) was examined by GFP fluorescence 4 days after transfection. Quantification shows dendritic spine number (B) was decreased by METTL3 knockdown in primary cortical neurons. For each neuron, dendritic segments with 100-200 μm in length beginning 100 μm from the cell body were selected for analysis (*n* = 39 neurons in each group). **(C-H)** Primary cortical neurons were infected by AAV-GFP-shRNA at DIV7. About 8–9 days after infection, primary neurons were extracted for immunoblot analysis (C) and immunofluorescence analysis (E, G). Quantifications shows decreased PSD95, but not Synaptophysin, after METTL3 depletion by immunoblot analysis (D) and immunofluorescence analysis (F), respectively. Quantification reveals reduced immunoreactivity of dendritic marker MAP 2 in shMettl3-infected neurons (H). (Data are means±SEM from at least 3 independent experiments, *p < 0.05, ***p < 0.001; B, D, F, H unpaired student’s t-test)

Dendritic spines as defined by protrusions of 0.5–5 μm in length with a clear neck and mushroom-like heads or a stubby shape [31] (Fig. 8 A) were examined by GFP fluorescence. Markedly reduced spine density was observed in primary neurons with METTL3 knockdown compared with control neurons (Fig. 8 A,B). Consistently, immunofluorescence analysis (Fig. 8 E,F) revealed reduced immunoreactivity of postsynaptic density protein 95 (PSD95). Interestingly, no changes in the immunoreactivity of presynaptic protein synaptophysin (Syp) was noted in primary neurons after METTL3 knockdown. Immunoblot analysis (Fig. 8 C,D) confirmed significantly reduced protein expression of PSD95 but not synaptophysin in primary neurons after METTL3 knockdown.

The existence of shortened or fragmented distal neurites was considered as a sign of neurite degeneration [39]. We observed an increase in the percentage of neurons with degenerative neurites in shMettl3-transfected primary neurons (Fig. S4 A,C). Meanwhile, the neurite number per soma was significantly reduced by METTL3 knockdown (Fig. S4 A,D). Similarly, immunofluorescence analysis revealed significant decrease in the protein level of dendritic marker MAP 2 in neurons after METTL3 knockdown (Fig. 8 G,H).

### Inhibition of oxidative stress and cell cycle events alleviates shMettl3-induced cell cycle abnormalities and neurodegenerative changes in primary neurons

**Fig. 9:**
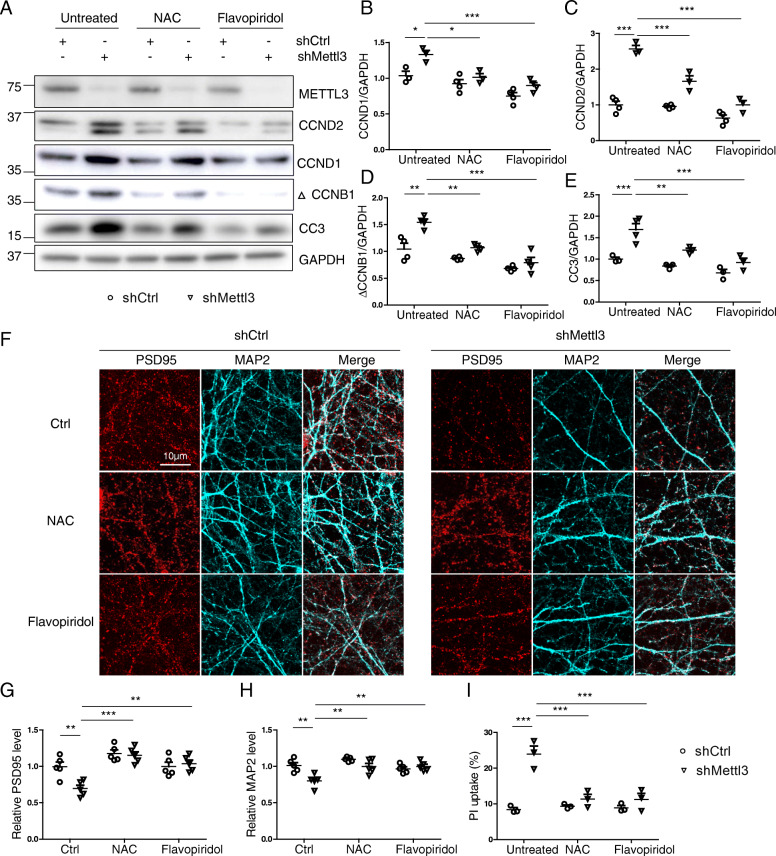
Inhibition of oxidative stress and cell cycle events alleviates shMettl3-induced cell cycle abnormalities and neurodegenerative changes in primary neurons. Primary cortical neurons were infected by AAV-GFP-shRNA at around DIV7. About 8–9 days after infection, primary neurons were used for analysis. Representative immunoblot (A) and quantitative analysis of CCND1 (B), CCND2 (C), CCNB1 (D) and cleaved caspase 3 (E) in primary neurons after AAV-shRNAs infection with/without co-treatment of 100 μM NAC or 40 nM flavopiridol. Representative immunofluorescence image (F) and quantitative analysis of PSD95 (G) and MAP 2 (H) in the neurites of primary cortical neurons after AAV-shRNAs infection with/without co-treatment of 100 μM NAC or 40 nM flavopiridol. **(I)** Cell death was measured by propidium iodide (PI) uptake in primary cortical neurons after AAV-shRNA infection with/without co-treatment of 100 μM NAC or 40 nM flavopiridol (Data are means±SEM from at least 3 independent experiments, *p < 0.05, **p < 0.01, ***p < 0.001; B-E, G-I one-way ANOVA with bonferroni’s correction)

To explore the causal relationship between METTL3 knockdown-induced oxidative stress, aberrant cell cycle events and degenerative changes in neurons, we used antioxidant N-acetyl cysteine (NAC) or cell cycle inhibitor flavopiridol to treat shMettl3-infected primary neurons. Flavopiridol treatment almost completely prevented METTL3 knockdown-induced abnormal increase in the protein levels of CCND1, CCND2 and △CCNB1, suggesting effective inhibition of METTL3 knockdown-induced aberrant cell cycle events (Fig. 9 A-D). Importantly, in this condition, METTL3 knockdown-induced caspase 3 (Fig. 9 A,E) activation and other neurodegenerative changes such as loss of postsynaptic PSD95 (Fig. 9 F,G), dendritic MAP 2 (Fig. 9 F,H) were also significantly inhibited. Interestingly, NAC also alleviated METTL3 knockdown-induced aberrant cell cycle events and caspase 3 activation (Fig. 9 A-E) along with restoration of postsynaptic PSD95 (Fig. 9 F,G) and dendritic MAP 2 (Fig. 9 F,H) in treated primary neurons. METTL3 knockdown-induced neuronal death, as indicated by propidium iodide (PI) uptake, was also alleviated by NAC and flavpiridol treatment (Fig. 9 I).

### Inhibition of m^6^A demethylation reduces shMettl3-induced deficits in primary neurons

**Fig. 10:**
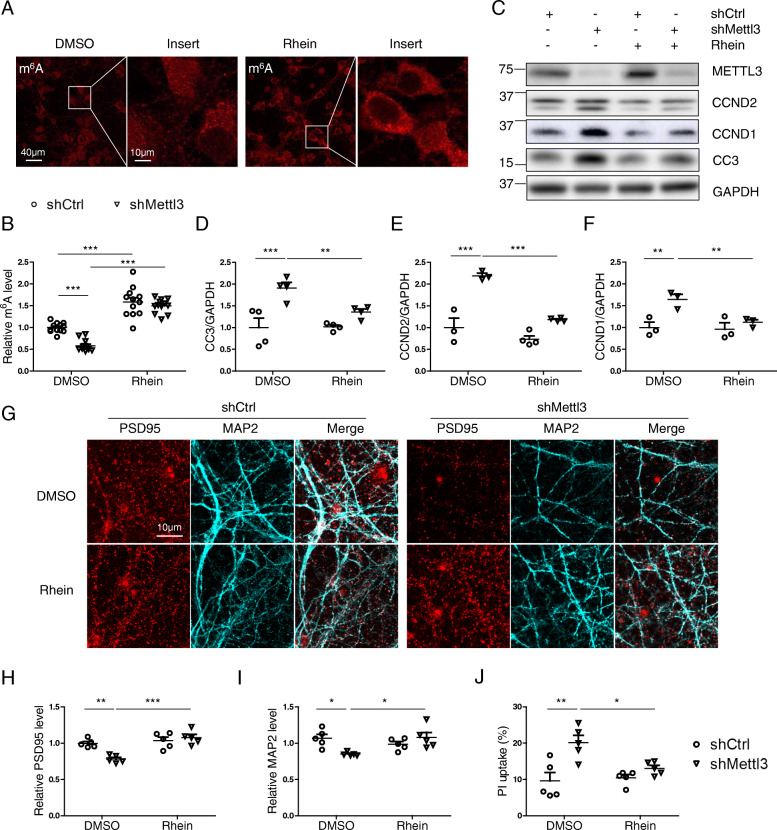
Inhibition of m^6^A demethylation reduces shMettl3-induced deficits in primary neurons. Primary cortical neurons were infected by AAV-GFP-shRNA at around DIV7. About 8–9 days after infection, primary neurons were used for analysis. Representative immunofluorescence image (**A**) and quantitative analysis of m^6^A (**B**) in the primary cortical neurons after AAV-shRNAs infection with/without co-treatment of 0.25 μM rhein (inhibitor for m^6^A demethylation) for 24 h. **(C-I)** Representative immunoblot (C) and quantitative analysis of cleaved caspase 3 (D), CCND2 (E) and CCND1 (F) in primary neurons after AAV-shRNAs infection with/without co-treatment of 0.25 μM rhein. (n = 3 in each group). Representative immunofluorescence image (G) and quantitative analysis of PSD95 (H) and MAP 2 (I) in the neurites of primary cortical neurons after AAV-shRNAs infection with/without co-treatment of Rhein. **(J)** Cell death was measured by propidium iodide (PI) uptake in primary cortical neurons after AAV-shRNA infection with/without co-treatment of Rhein (Data are means±SEM from at least 3 independent experiments, *p < 0.05, **p < 0.01, ***p < 0.001; B, D-F, H-J one-way ANOVA with bonferroni’s correction)

Next we investigated whether restoration of m^6^A modification by treatment of rhein, a natural product and small-molecule inhibitor of FTO demethylase [60], could rescue METTL3 depletion-induced reduced m^6^A modification and neuronal deficits. As reported previously [61], basal levels of neuronal m^6^A modification was elevated by rhein treatment (Fig. 10 A). METTL3 knockdown-induced m^6^A decrease in neurons was blocked by rhein treatment (Fig. 10 B). Interestingly, inhibition of m^6^A demethylation effectively alleviated dysregulation of cell cycle genes (i.e., CCND 1 and 2) and the cleavage/activation of caspase 3 induced by METTL3 knockdown in primary neurons (Fig. 10 C-F). METTL3 knockdown-induced other degenerative changes such as loss of postsynaptic PSD95 (Fig. 10 G,H) and dendritic MAP 2 (Fig. 10 G,I) were also restored by rhein. METTL3 knockdown-induced neuronal loss was also partially rescued by rhein treatment as measured by PI uptake assay (Fig. 10 J).

### METTL3 overexpression rescues Aβ-induced synaptic toxicity both in vitro and in vivo

**Fig. 11:**
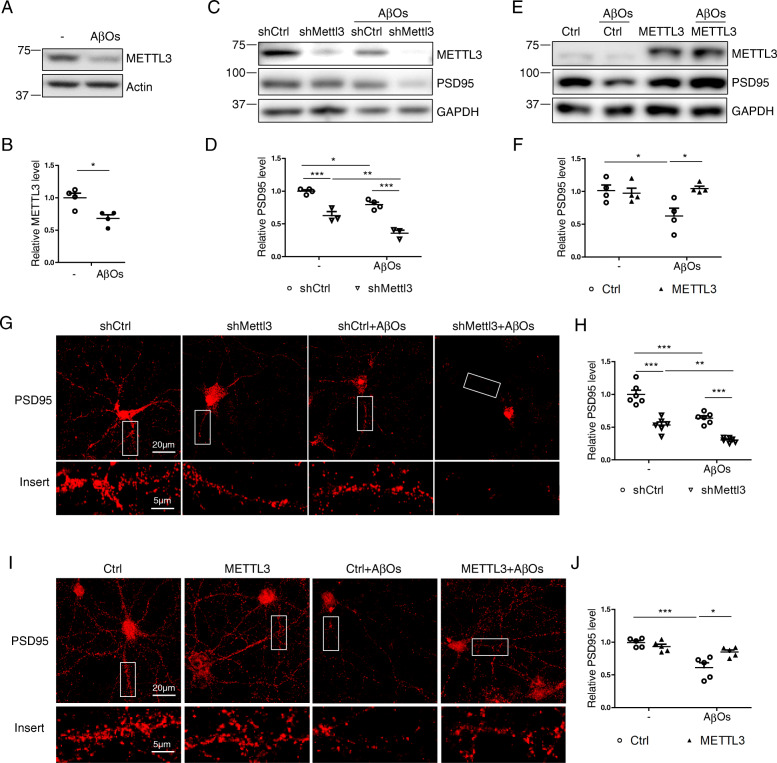
Manipulated METTL3 expression impacts AβO-induced PSD95 loss in primary neurons. (**A-B**) Representative immunoblot (A) and quantitative analysis (B) of METTL3 protein expression in primary rat cortical neurons treated by 2.5 μM AβOs at DIV14 for 3 days. (C-H) Primary rat cortical neurons were infected by AAV-shMettl3 (C, D, G) or AAV-METTL3 (E, F, H) at ~DIV7 and challenged by 2.5 μM AβOs at DIV14 for 3 days. Representative immunoblot (C, E) and quantification analysis (D, F) of METTL3 and PSD95 at DIV17–18 were shown. Representative immunofluorescence images of PSD95 in neurites were shown (G, I) and relative PSD95 immunoreactivity was quantified (H, J). (Data are means±SEM from at least 3 independent experiments, *p < 0.05, **p < 0.01, ***p < 0.001; B, unpaired student’s t-test; D, F, H, J one-way ANOVA with bonferroni’s correction)

It was suggested that soluble Aβ oligomers (AβO) are the culprit of AD which cause synaptic loss and neuronal dysfunction in AD [62]. Interestingly, AβO treatment caused significantly reduced METTL3 expression in rat primary cortical neurons (Fig. 11 A,B). To assess how METTL3 expression impacts Aβ-induced synaptic deficits, METTL3 expression was manipulated in primary cortical neurons which were then treated with 2.5 μM AβOs for 3 days. As previously reported, immunoblot analysis demonstrated that AβOs treatment led to significant reduction of PSD95 protein level in shCtrl-infected neurons, which was dramatically exacerbated by METTL3 knockdown (Fig.11 C,D). This was corroborated by immunostaining analysis which also revealed that AβOs-induced PSD95 reduction in shCtrl-infected neurons was further exacerbated by METTL3 knockdown (Fig. 11 G,H). On the contrary, METTL3 overexpression significantly alleviates AβOs-induced PSD95 reduction by both immunoblot analysis (Fig. 11 E,F) and immunofluorescence analysis (Fig. 11 I,J).

**Fig. 12:**
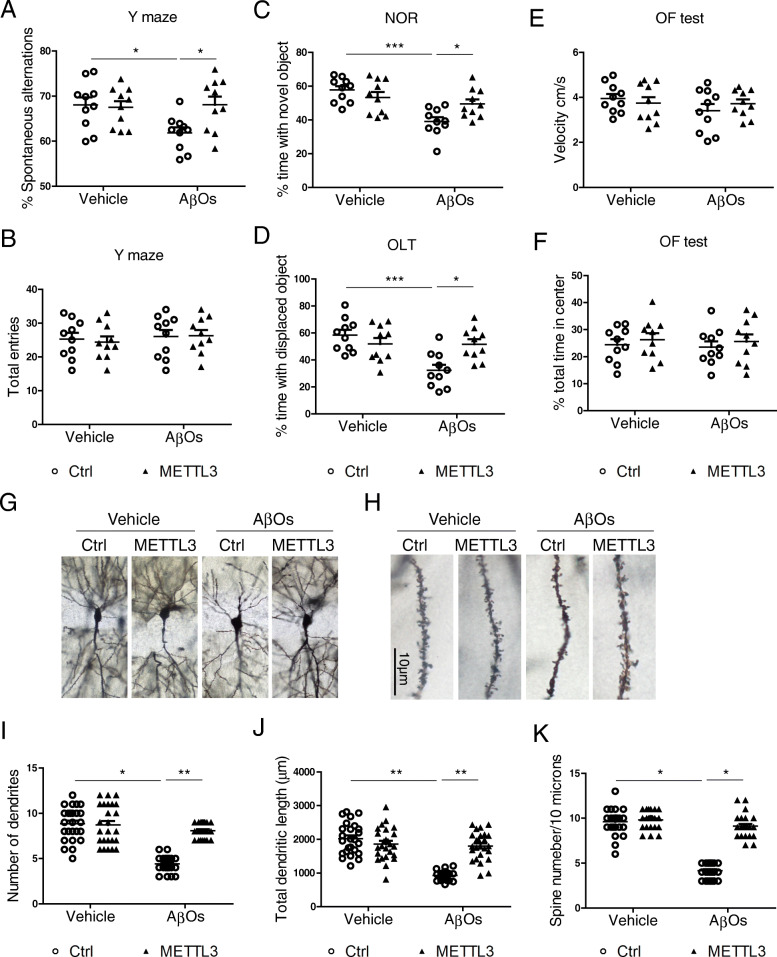
METTL3 overexpression rescues AβO-induced cognitive impairment and synaptic deficits in vivo. WT C57BL/6 mice receiving bilaterally stereotaxic injections of AAV-METTL3/AAV-Ctrl into the hippocampus at ~ 2 months of age were administrated with AβO preparations 2 weeks later. Behavioral tests were performed 2 week after AβO treatment. Cognitive functions were measured by Y maze task (A-B), novel object recognition test (NOR) (C) and object location test (OLT) (D). Locomotor function (E) and exploring activity (F) were measured by open field test (OF) (*n* = 10 mice/group, sex matched). (**G-K**) Dendrites (G) and spines (H) in the infused hippocampal areas were examined by Golgi stain. Number of dendrites (I), total length of dendrites (J) and number of dendritic spines (K) were measured. (*n* = 25 neurons from 5 mice in each group) (*p < 0.05, **p < 0.01, ***p < 0.001; A-F, I-K, one-way ANOVA with bonferroni’s correction)

To assess whether METTL3 overexpression rescues AβOs-induced synaptic damage and cognition/memory deficits in vivo, we stereotactically injected AAV-METTL3 into the CA1 region of 9–10 week old C57BL/6 mice (Fig.S6 A,B) and AβOs were injected into the same site two weeks later. After an additional two weeks, these mice were subject to cognitive tests before sacrifice. As we reported previously [35, 36], AβOs treatment led to significant cognitive deficits as determined by Y-maze task (Fig. 12 A,B), novel object recognition test (Fig. 12 C) and object location test (Fig. 12 D). Importantly, METTL3 overexpression effectively rescued the AβOs-induced cognitive deficits in these mice. Locomotor function (Fig. 12 E) and exploring activities (Fig. 12 F) were unaffected by AβOs and METTL3 as indicated by open field tests. Consistently, golgi staining revealed that AβOs-induced loss of dendrites and spines in the hippocampus was almost completely prevented in METTL3 overexpression mice (Fig. 12 G-K).

## Discussion

Despite relatively high RNA m^6^A level in the brain [58], study of m^6^A modification in the brain is a nascent field, and the significance of this epigenetic mark in brain development and neuronal function is just beginning to be appreciated. In this study, we found significantly reduced RNA m^6^A modification in the susceptible pyramidal neurons but increased RNA m^6^A in glial cells in the hippocampus and cortex of AD patients. Significantly reduced protein expression of m^6^A writers (METTL3, METTL14 and WTAP), eraser (FTO) and reader (YTHDF1) accompanied by reduced nuclear distribution of METTL3/14 in the pyramidal neurons were also found in AD brain. While these findings in human AD brain are correlational, it helps establish the pathophysiological relevance of the study into METTL3 dysregulation in AD. Importantly, METTL3 and METTL14 levels were reduced in the brain of MCI patients, a prodromal stage of AD, suggesting an early role of dysregulated RNA m^6^A modification during the course of AD. AD is characterized by cognitive impairment and progressive neurodegeneration, which is believed to be caused by early synaptic damage and eventually selective neuronal loss in AD-affected brain areas [63]. Signifying the critical role of the appropriate m^6^A equilibrium in the hippocampal function in adult mice, reduced m^6^A deposition induced by METTL3 knockdown in the hippocampus of adult mice caused significant cognitive/memory impairment. In exploring the brain pathology that underlie the abnormal memory decline after METTL3 knockdown, we found significant dendritic spine and synaptic abnormalities and extensive neuronal death in the hippocampus, all features of AD. These abnormal neuronal deficits and death are probably specific effects of reduced overall m^6^A modification since they could be rescued in primary neurons by treatment of rhein, a small-molecule inhibitor of FTO demethylase that restored m^6^A modification levels. Additional experiments demonstrated that treatment of Aβ oligomers caused reduced METTL3 expression in primary neurons and overexpression of METTL3 rescued Aβ oligomers-induced synaptic deficits both in vitro and in vivo. More importantly, overexpression of METTL3 rescued Aβ oligomers-induced cognitive deficits in adult mice.

A major finding of this study is significantly reduced neuronal RNA m^6^A modification along with significantly reduced expression of m^6^A regulators such as METTL3 in AD brain. The finding of significantly reduced METTL3 expression in MCI patients underscores an early and critical role of m^6^A dysregulation in AD pathogenesis. The lack of changes in other m^6^A regulators during MCI phase suggests that METTL3 reduction may be an initiating factor in m6A dysregulation which lead to reduction in other m6A regulators at a later stage during the course of AD because METTL3 and METTL14 form a heterodimeric methyltransferase complex and METTL3 knockdown leads to METTL14 reduction [64]. Similarly, changes in other m6A regulators, especially m6A erasers such as FTO reduction could be an adaptive response to early changes in METTL3 and/or 14. These will need to be investigated in future studies into the role of m^6^A dysregulation in the pathogenesis of AD. Nevertheless, our finding of reduced METTL3 expression in AD is consistent with the recent report of reduced METTL3 expression in AD based on the expression profile study using a public RNA-seq dataset [65] which suggested accumulation of METTL3 in the insoluble fraction may play a role. Our study further suggested that abnormal redistribution of m^6^A-related proteins between nucleus and cytosol may also contribute to the decrease of neuronal m^6^A modification in AD. A nuclear anchoring protein ZC3H13 in RNA m^6^A methylation complex was identified recently [66], highlighting that nuclear localization of m^6^A regulatory complex is critical for m^6^A modification and its biological function. In this regard, more dramatic reduction of METTL3 and METTL14 writers in the nucleus was noted in AD neurons. Consistent with reduced METTL3 in AD brain, we found treatment of Aβ oligomers caused reduced METTL3 expression in primary neurons. However, it remains elusive how m^6^A modification and METTL3 expression change in the brain of APP transgenic models because conflicting results were reported: Han et al. reported increased global m^6^A levels in total RNA in the cortex and hippocampus of APP/PS1 mice at 9 months of age using an m^6^A RNA methylation quantification kit [67]. They also reported an increased mRNA expression of METLL3 in these mice [67]. On the contrary, a more recent study demonstrated significantly reduced METLL3 expression along with reduced m^6^A levels in poly(A) mRNA in 6 months old 5xFAD mice measured by LC-MS/MS [68]. It remains to be determined what caused this discrepancy which could be due to different sample preparation (total RNA vs. mRNA) and/or methods (antibody based colorimetric method vs. LC-MS/MS). Model-specific effects may also be involved since neuronal death was only observed in the 5xFAD model.

Another major finding is that METTL3 knockdown caused cognitive deficits and neurodegeneration which support a critical role of METTL3 reduction in mediating major deficits related to AD. This was further strengthened by the findings that METTL3 overexpression fully rescued Aβ oligomers-induced synaptic deficits and cognitive deficits in vivo. We also observed other neuronal abnormalities related to AD pathogenesis by shMettl3-induced m^6^A dysregulation in the adult hippocampus, which could shed light on the underlying neurodegeneration mechanism. Pyramidal neurons are postmitotic and quiescent cells. However, aberrant cell cycle events, likely induced by increased DNA damage, has long been noted as a prominent feature in many of the susceptible pyramidal neurons which is considered to result in their eventual demise through apoptosis and contribute to the degeneration of neuronal tissue in AD [53, 69]. Most recently, integration of epigenetic and transcriptomic data demonstrates a pro-apoptotic reactivation of the cell cycle in post-mitotic AD neurons [6]. In this study, we found METTL3 knockdown caused increased oxidative stress and DNA damage, aberrant cell cycle events and apoptotic activation in pyramidal neurons in the adult hippocampus in vivo after METTL3 knockdown. While a causal relationship between these abnormal changes (oxidative stress and aberrant cell cycle events) and eventual cell death remains to be established in vivo since the possibility that these abnormal changes could simply be due to the degenerative changes of neurons during death has not been ruled out, shMettl3-induced dysregulation of cell cycle genes was rescued by restoration of m^6^A after rhein treatment, accompanied by blocked activation of apoptosis and neuronal deficits in vitro. It highlights that METTL3 depletion reduces m^6^A modification and thus induces dysregulation of cell cycle genes, activation of apoptotic pathway and AD-related neuronal deficits. Moreover, inhibition of cell cycle events by flavopiridol treatment alleviates shMettl3-induced dysregulation of cell cycle genes and neuronal deficits and apoptosis, indicating a causative role of dysregulation of cell cycle genes in shMettl3-induced AD-related neuronal deficits and death. How do pyramidal neurons commit to an unscheduled cell cycle after METTL3 knockdown that lead to their doomed fate? It has been shown that there is an enrichment of genes in m^6^A-tagged transcripts related to the cell cycle, stem cells and neuronal differentiation and m^6^A methylation of mRNAs related to the cell cycle regulates their turnover and changes the temporal specification and cell cycle progression [24, 70]. We also confirmed m^6^A methylation in the G1 phase regulator CCND2 and increased levels of mRNA and protein of CCND2 in the hippocampus or primary neurons after METTL3 knockdown. It is possible that reduced m^6^A deposition after METTL3 knockdown leads to uncoordinated changes in the expression of cell cycle regulators including CCND1/2 and resulted in unscheduled cell cycle re-entry that leads to apoptosis. Our data also suggested that oxidative stress may contribute to aberrant cell cycle-induced neuronal apoptosis since antioxidant NAC alleviates shMettl3-induced dysregulation of cell cycle genes and protects against the subsequent apoptosis activation and neuronal damage. In this regard, it is noted that cell cycle and oxidative stress can be team players in neurodegenerative disorders and oxidative stress can lead to cell cycle abnormalities through DNA damage response [71]. Interestingly, RNA m^6^A methylation regulates the ultraviolet-induced DNA damage response [64], indicating the protective role of RNA m^6^A modification in DNA repair. Therefore, it is possible that the accumulation of DNA damage as evidenced by γH2AX due to increased oxidative stress and a lack of proper DNA repair after METTL3 knockdown also contributes to the unscheduled cell cycle re-entry in these susceptible pyramidal neurons.

Contrary to significantly reduced neuronal RNA m^6^A modification in the hippocampus and cortex of AD patients, significantly increased RNA m^6^A modification was noted in the microglia and astrocytes in these brain areas. This probably explains the lack of changes in the overall levels of m^6^A deposition in AD brain measured by LC-MS/MS. It is unclear what caused differential RNA m^6^A deposition between neurons and glial cells in AD. Its functional significance is also not clear. Inflammation occurs in pathologically vulnerable regions of the AD brain and contributes to AD pathogenesis as demonstrated in animal models and clinical studies [72]. Activated microglia and astrocytes were found in the hippocampus after METTL3 knockdown. However, since only neuronal cells were infected by AAV shRNA in our mouse model, enhanced inflammation in the mouse hippocampus after METTL3 knockdown was probably a pathological response to shMettle3-induced synaptic damage and cell death instead of being the direct effect of reduced glial m^6^A. Therefore, it is still possible that increased m^6^A deposition in glial cells could contribute to the enhanced inflammation and disease development which should be further explored.

There are methodological limitations to this study. First, m^6^A antibodies can cross-react with N^6^, 2′-O-dimethyladenosine (m^6^Am), a modification found adjacent to the N^7^-methylguanosine (m^7^G) cap at the first nucleotide of certain mRNAs [73]. However, the pathological roles of neuronal METTL3 reduction and RNA m^6^A dysregulation in AD are unlikely affected by this limitation, since a cap-specific adenosine methyltransferase (CAPAM) is responsible for m^6^Am modification [74]. The effect of m^6^Am modification on m^6^A measurement is unknown in this study and potential function of m^6^Am modification in AD needs further exploration. Second, cell-type specific changes of m^6^A in AD were only demonstrated by immunocytochemistry which depends on the specificity of the antibody. This should be confirmed by different methods with large number of human samples. However, it’s technically difficult to prepare intact neurons in sufficient amount from postmortem human brains for m^6^A measurement by LC-MS/MS. Lastly, the in vitro and in vivo models used in the current study involved acute treatment of AβOs. It would be of interest to determine the impact of METTL3 in aged mouse models carrying either mutant APP or tau.

## Conclusion

Overall, our study demonstrated that RNA m^6^A modification has an essential role in maintaining synaptic structure and neuronal survival and function in adult neurons in the hippocampus, which has broader implication in brain health. More specifically, we demonstrated that the m^6^A-related epitranscriptomic dysregulation contributes to the neurodegeneration and pathogenesis of AD, which opens new avenues of investigation into the pathophysiological function of m^6^A in the neurodegenerative diseases and how it becomes dysregulated during AD. Our study also suggested that METTL3-mediated m6A dysregulation may be a therapeutic target for AD.

## Supplementary Information

**Additional file 1: Supplementary Fig. 1.** Characterizations of AD brain tissues and negative controls for immunostaining of m6A modifications and its regulators in brain tissues. (A-C) Representative images of pTau (AT8) immunohistochemistry (A) and immunoblot analysis (B) in AD. Hyper phosphorylated Tau exists in all of the AD hippocampal cases (A) and significantly elevated pTau (AT8) protein level was noted in quantitative analysis (C). (D) Normal human hippocampal sections were treated with Rnase or Dnase overnight before stained for m6A using rabbit m6A antibody (SYSY, synaptic systems). Immunoreactivities of m6A modification were decreased in hippocampal tissue after Rnase treatment, indicating m6A modifications exist in in RNA. No change in m6A immunoreactivity was observed in hippocampal tissue after Dnase treatment. Negative control experiments were performed without primary antibody during Rnase and Dnase treatment and no cellular structure was noted. (E-F) Negative control experiments were performed without primary antibody in immunostaining for m6A modification (E) and m6A regulators in human AD and control brain cases. (n = 6, *p < 0.05, B, unpaired student’s t-test).

**Additional file 2: Supplementary Fig. 2.** m6A is increased in astrocytes in AD hippocampus, but limited colocalization of m6A with Iba1 in AD was observed. (A-B) Colocalization of m6A (Novus, A; SYSY, B) with astrocyte marker GFAP (Thermofisher, A; MP Biomedicals, B) in hippocampal tissues from AD and control brains. (C) Quantification revealed that m6A immunoreactivity was increased in astrocytes in AD hippocampal tissues compared with control. (D) AD and control hippocampal sections were stained for m6A (Novus) and Iba1. Only some colocalization of m6A and Iba1 was observed in AD and control hippocampal sections. (E) Negative control experiments were performed without primary antibody in immunostaining for m6A modification in human brain cases (n = 5–6 in each group, *p < 0.5, C, unpaired student’s t-test).

**Additional file 3: Supplementary Fig. 3.** Validation of intracranial injection into hippocampus by needle track and induced neuroinflammation by METTL3 depletion in mouse hippocampus. (A) A representative image of needle track (arrow) of AAV-injected mice was shown. GFP immunoreactivity was detected in area adjacent to the needle tack. NeuN staining revealed severe neuronal loss around injected areas only in AAV-shMettl3 injected mice but not AAV-shCtrl injected mice. (B-E) Representative images of immunohistochemistry for Iba1 (B) and GFAP (Thermofisher, D) in hippocampal CA1/2 or CA3 areas in shRNA-injected mice and their quantification (C for astrocyte and E for microglia) analysis showed that METTL3 knockdown caused neuroinflammation in mouse hippocampus. (n = 4–7, *p < 0.5, **p < 0.01; C, E, unpaired student’s t-test).

**Additional file 4: Supplementary Fig. 4.** METTL3 depletion leads to neurite degeneration in primary neurons. (A-D) GFP-shRNA was transfected into primary cortical neurons at DIV 9–12 using Lipofectamine 2000 according to manufacturer’s instruction. Then neuronal cultures were used in following analysis 4 days after transfection. Representative images of immunofluorescence for METTL3 (A) and quantification of METTL3 immunoreactivity (B) in positively-transfected (GFP) neurons showed that GFP-shMettl3 transfection efficiently knockdown the endogenous METTL3 in neurons (n = 11–15 neurons). Analysis of neuronal morphology based on GFP fluorescence showed increased percentage of neurons with abnormal neurites (C) and decreased neurite numbers per neuron (D) in METTL3 depleted neurons (n = 183–210 neurons). (*p < 0.5, **p < 0.01; B-D, unpaired student’s t-test).

**Additional file 5: Supplementary Fig. 5.** Statistical analysis of relative changes in protein levels between shMettl3 and shCtrl groups (i.e., shMettl3/shCtrl ratio) in response to NAC, Flavopiridol or Rhein based on data presented in Fig. 9 and Fig. 10. (A-D) shMettl3-induced significant elevations of CCND2, △CCNB1 and CC3 (i.e., shMettl3/shCtrl ratio significantly greater than 1) or reduction of PSD95 and MAP 2 (shMettl3/shCtrl ratio significantly less than 1) are rescued by NAC, Flavopiridol or Rhein treatment. (*p < 0.5, **p < 0.01; A-D, unpaired student’s t-test).

**Additional file 6: Supplementary Fig. 6.** Validation of AAV-mediated METTL3 overexpression in mouse hippocampus. GFP expression of AAV-Ctrl (A) was detected by fluorescence in hippocampal area and (B) METTL3 overexpression was confirmed by western blot in AAV-METTL3 mouse hippocampus.

## Data Availability

All the data supporting the conclusions of the current study are presented in the figures and they are available from the corresponding authors upon reasonable request. There are no restrictions on data availability. Source data are provided with this paper.

## Abbreviations

AAV: adeno-associated virus
AD: Alzheimer’s disease
ALKBH5: α-ketoglutarate-dependent dioxygenase alkB homolog 5
CCNB1: cyclin B1
CCND1: cyclin-D1
CCND2: cyclin-D2
CDKN1C: cyclin dependent kinase inhibitor 1C
CWRU: Case Western Reserve University
eGFP: enhanced green fluorescent protein
FTO: fat mass and obesity associated protein
IP: immunoprecipitation
m^6^A: N6-methyladenosine
MCI: mild cognitive impairment
METTL3: methyltransferase like 3
NAC: N-acetyl cysteine
NOR: novel object recognition
OF: open field
OLT: object location test
PCNA: proliferating cell nuclear antigen
PI: propidium iodide
PSD95: postsynaptic density protein 95
RIPA: radioimmunoprecipitation assay
ROS: reactive oxygen species
SAT: spontaneous alternation task
SEM: standard error of the mean
shCtrl: scramble shRNA as a negative control
shMettl3: METTL3-targetting shRNA
Syp: synaptophysin
WTAP: Wilms Tumor 1 Associated Protein
