# Supplementary figures and images for: METTL3-dependent RNA m6A dysregulation contributes to neurodegeneration in Alzheimer’s disease through aberrant cell cycle events
Source: Mol Neurodegener. 2021 Sep 30;16:70. doi: 10.1186/s13024-021-00484-x (PMC8482683; doi:10.1186/s13024-021-00484-x)

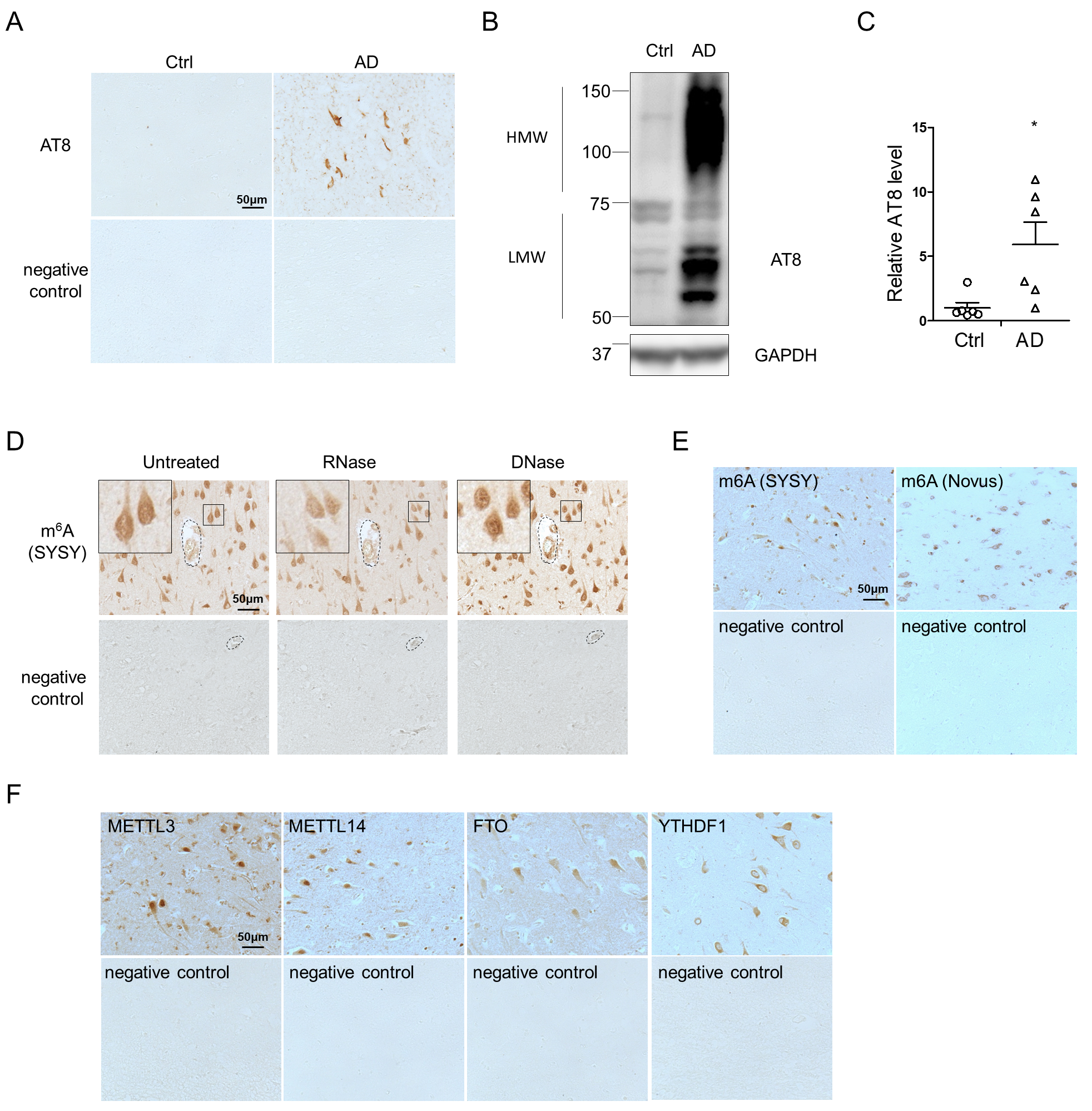

Supplement: Supplementary file 1 — Additional file 1: Supplementary Fig. 1. Characterizations of AD brain tissues and negative controls for immunostaining of m6A modifications and its regulators in brain tissues. (A-C) Representative images of pTau (AT8) immunohistochemistry (A) and immunoblot analysis (B) in AD. Hyper phosphorylated Tau exists in all of the AD hippocampal cases (A) and significantly elevated pTau (AT8) protein level was noted in quantitative analysis (C). (D) Normal human hippocampal sections were treated with Rnase or Dnase overnight before stained for m6A using rabbit m6A antibody (SYSY, synaptic systems). Immunoreactivities of m6A modification were decreased in hippocampal tissue after Rnase treatment, indicating m6A modifications exist in in RNA. No change in m6A immunoreactivity was observed in hippocampal tissue after Dnase treatment. Negative control experiments were performed without primary antibody during Rnase and Dnase treatment and no cellular structure was noted. (E-F) Negative control experiments were performed without primary antibody in immunostaining for m6A modification (E) and m6A regulators in human AD and control brain cases. (n = 6, *p < 0.05, B, unpaired student’s t-test). [file 13024_2021_484_MOESM1_ESM.tif]

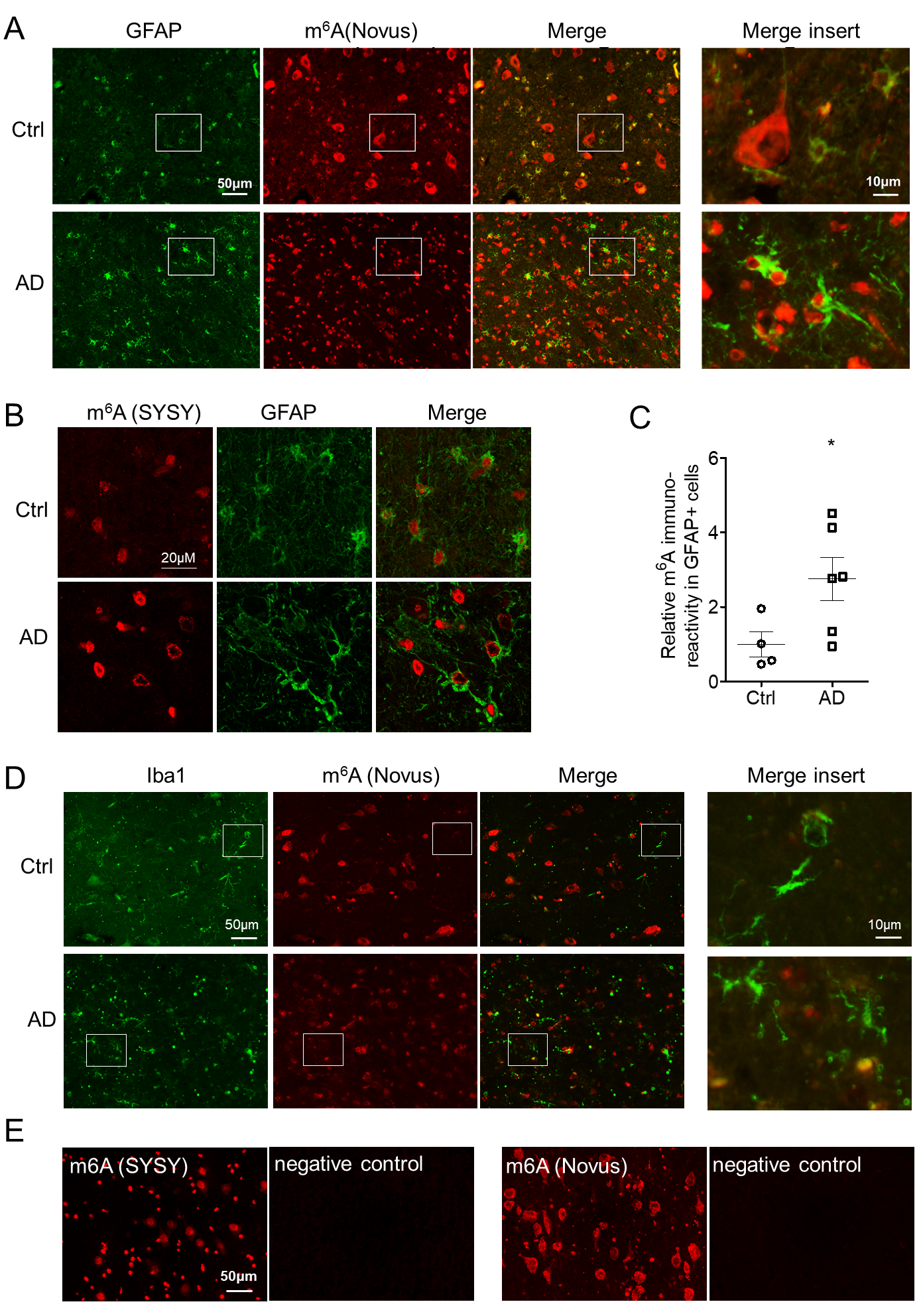

Supplement: Supplementary file 2 — Additional file 2: Supplementary Fig. 2. m6A is increased in astrocytes in AD hippocampus, but limited colocalization of m6A with Iba1 in AD was observed. (A-B) Colocalization of m6A (Novus, A; SYSY, B) with astrocyte marker GFAP (Thermofisher, A; MP Biomedicals, B) in hippocampal tissues from AD and control brains. (C) Quantification revealed that m6A immunoreactivity was increased in astrocytes in AD hippocampal tissues compared with control. (D) AD and control hippocampal sections were stained for m6A (Novus) and Iba1. Only some colocalization of m6A and Iba1 was observed in AD and control hippocampal sections. (E) Negative control experiments were performed without primary antibody in immunostaining for m6A modification in human brain cases (n = 5–6 in each group, *p < 0.5, C, unpaired student’s t-test). [file 13024_2021_484_MOESM2_ESM.tif]

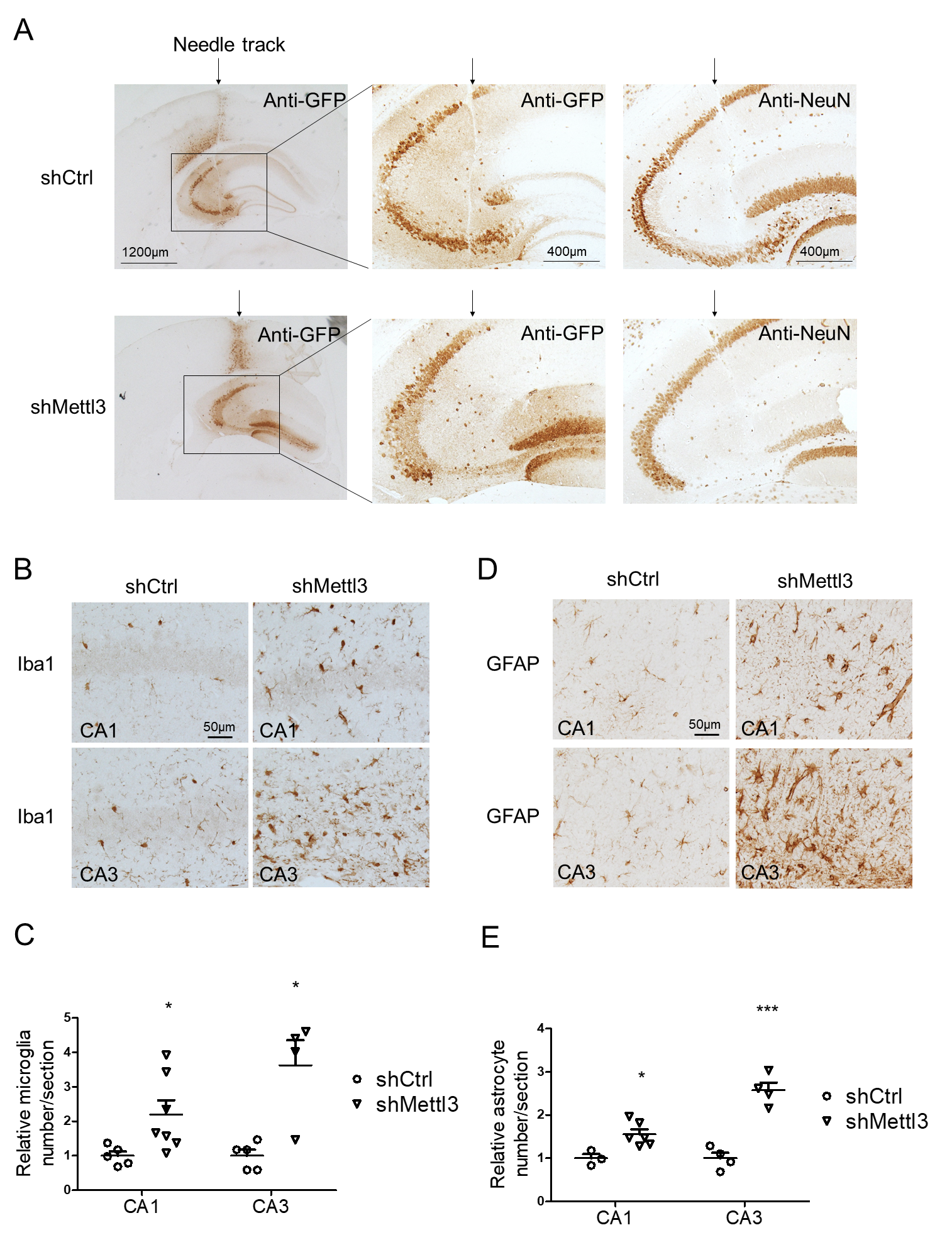

Supplement: Supplementary file 3 — Additional file 3: Supplementary Fig. 3. Validation of intracranial injection into hippocampus by needle track and induced neuroinflammation by METTL3 depletion in mouse hippocampus. (A) A representative image of needle track (arrow) of AAV-injected mice was shown. GFP immunoreactivity was detected in area adjacent to the needle tack. NeuN staining revealed severe neuronal loss around injected areas only in AAV-shMettl3 injected mice but not AAV-shCtrl injected mice. (B-E) Representative images of immunohistochemistry for Iba1 (B) and GFAP (Thermofisher, D) in hippocampal CA1/2 or CA3 areas in shRNA-injected mice and their quantification (C for astrocyte and E for microglia) analysis showed that METTL3 knockdown caused neuroinflammation in mouse hippocampus. (n = 4–7, *p < 0.5, **p < 0.01; C, E, unpaired student’s t-test). [file 13024_2021_484_MOESM3_ESM.tif]

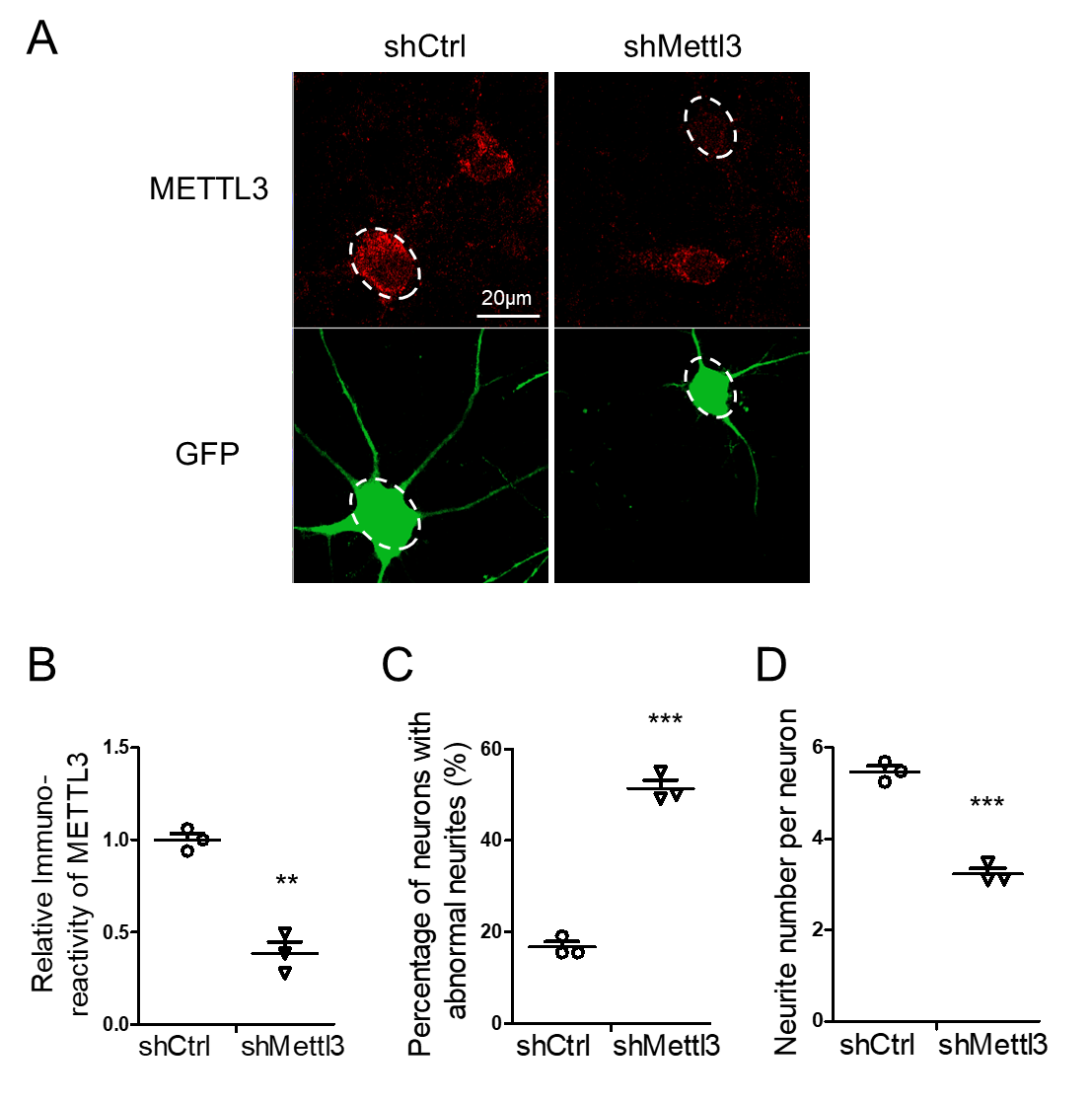

Supplement: Supplementary file 4 — Additional file 4: Supplementary Fig. 4. METTL3 depletion leads to neurite degeneration in primary neurons. (A-D) GFP-shRNA was transfected into primary cortical neurons at DIV 9–12 using Lipofectamine 2000 according to manufacturer’s instruction. Then neuronal cultures were used in following analysis 4 days after transfection. Representative images of immunofluorescence for METTL3 (A) and quantification of METTL3 immunoreactivity (B) in positively-transfected (GFP) neurons showed that GFP-shMettl3 transfection efficiently knockdown the endogenous METTL3 in neurons (n = 11–15 neurons). Analysis of neuronal morphology based on GFP fluorescence showed increased percentage of neurons with abnormal neurites (C) and decreased neurite numbers per neuron (D) in METTL3 depleted neurons (n = 183–210 neurons). (*p < 0.5, **p < 0.01; B-D, unpaired student’s t-test). [file 13024_2021_484_MOESM4_ESM.tif]

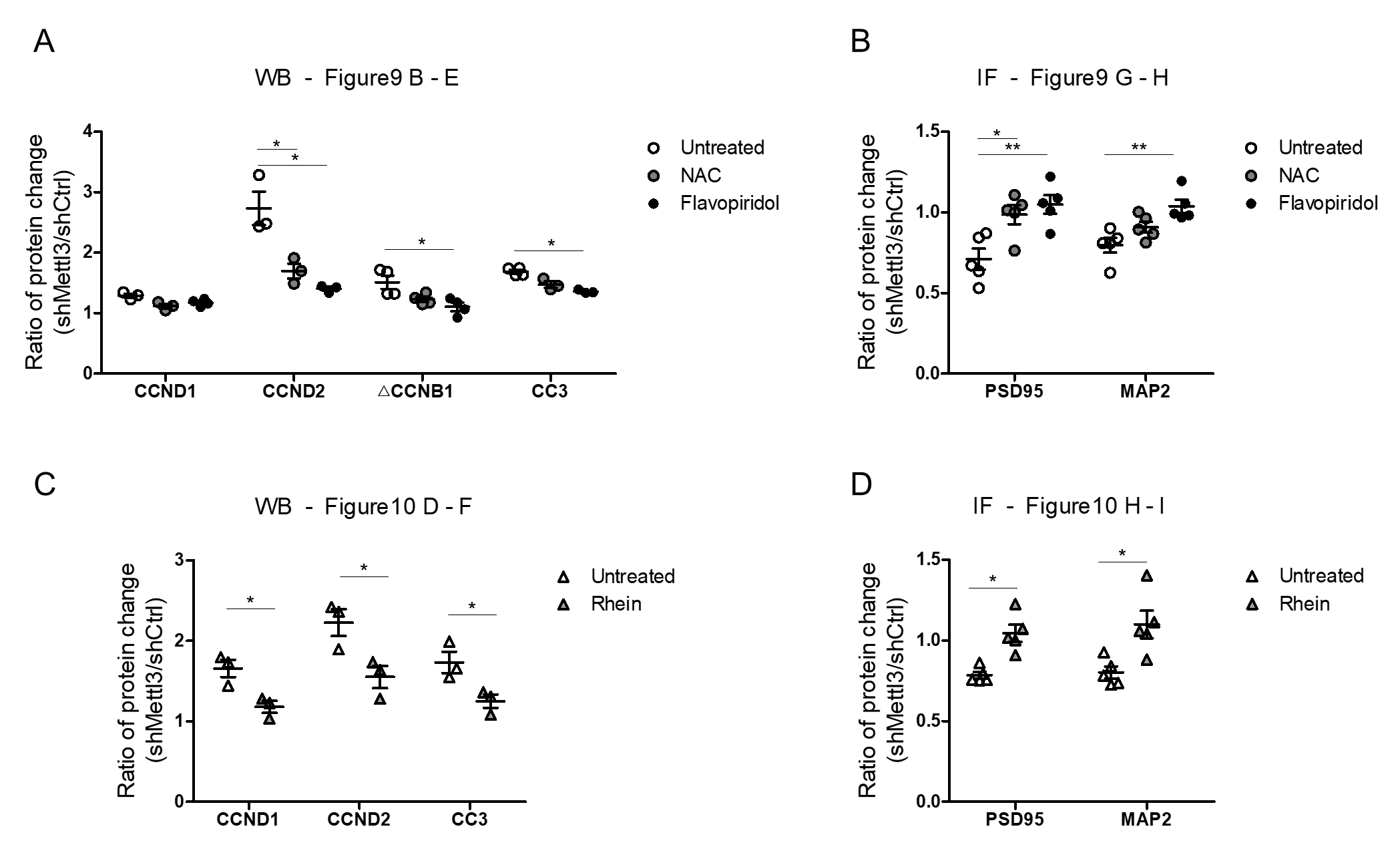

Supplement: Supplementary file 5 — Additional file 5: Supplementary Fig. 5. Statistical analysis of relative changes in protein levels between shMettl3 and shCtrl groups (i.e., shMettl3/shCtrl ratio) in response to NAC, Flavopiridol or Rhein based on data presented in Fig. 9 and Fig. 10. (A-D) shMettl3-induced significant elevations of CCND2, △CCNB1 and CC3 (i.e., shMettl3/shCtrl ratio significantly greater than 1) or reduction of PSD95 and MAP 2 (shMettl3/shCtrl ratio significantly less than 1) are rescued by NAC, Flavopiridol or Rhein treatment. (*p < 0.5, **p < 0.01; A-D, unpaired student’s t-test). [file 13024_2021_484_MOESM5_ESM.tif]

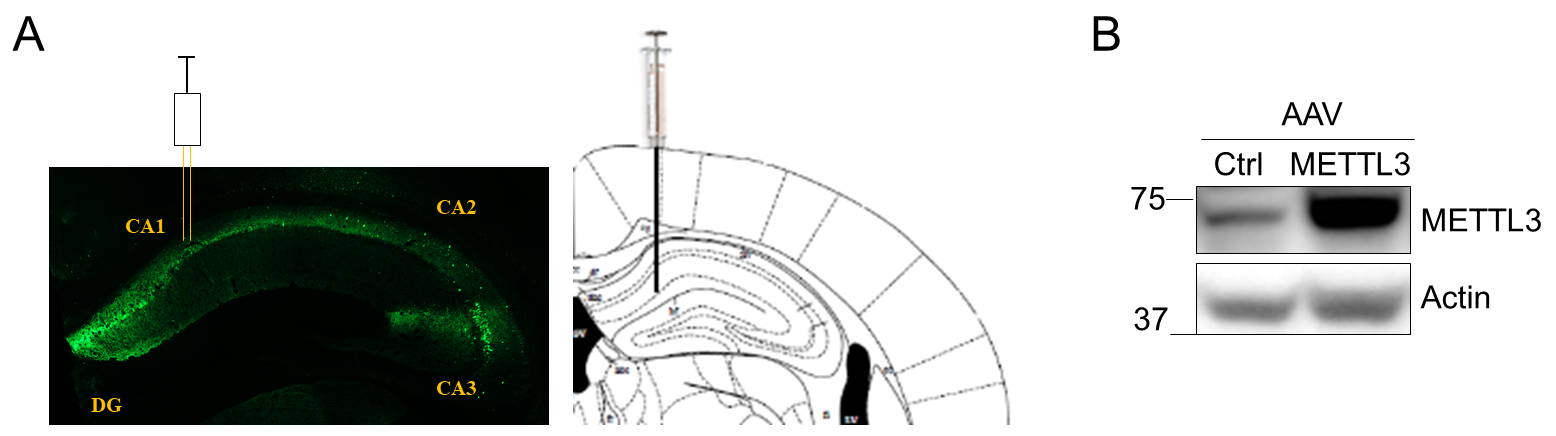

Supplement: Supplementary file 6 — Additional file 6: Supplementary Fig. 6. Validation of AAV-mediated METTL3 overexpression in mouse hippocampus. GFP expression of AAV-Ctrl (A) was detected by fluorescence in hippocampal area and (B) METTL3 overexpression was confirmed by western blot in AAV-METTL3 mouse hippocampus. [file 13024_2021_484_MOESM6_ESM.tif]
